# MetaTron: advancing biomedical annotation empowering relation annotation and collaboration

**DOI:** 10.1186/s12859-024-05730-9

**Published:** 2024-03-14

**Authors:** Ornella Irrera, Stefano Marchesin, Gianmaria Silvello

**Affiliations:** Department of Information Engineering, University of Padova, Padua, Italy

**Keywords:** Digital health, Biomedical annotation tool, Relation extraction

## Abstract

**Background:**

The constant growth of biomedical data is accompanied by the need for new methodologies to effectively and efficiently extract machine-readable knowledge for training and testing purposes. A crucial aspect in this regard is creating large, often manually or semi-manually, annotated corpora vital for developing effective and efficient methods for tasks like relation extraction, topic recognition, and entity linking. However, manual annotation is expensive and time-consuming especially if not assisted by interactive, intuitive, and collaborative computer-aided tools. To support healthcare experts in the annotation process and foster annotated corpora creation, we present *MetaTron*. *MetaTron* is an open-source and free-to-use web-based annotation tool to annotate biomedical data interactively and collaboratively; it supports both mention-level and document-level annotations also integrating automatic built-in predictions. Moreover, *MetaTron* enables relation annotation with the support of ontologies, functionalities often overlooked by off-the-shelf annotation tools.

**Results:**

We conducted a qualitative analysis to compare *MetaTron* with a set of manual annotation tools including *TeamTat*, *INCEpTION*, *LightTag*, *MedTAG*, and *brat*, on three sets of criteria: technical, data, and functional. A quantitative evaluation allowed us to assess *MetaTron* performances in terms of time and number of clicks to annotate a set of documents. The results indicated that *MetaTron* fulfills almost all the selected criteria and achieves the best performances.

**Conclusions:**

*MetaTron* stands out as one of the few annotation tools targeting the biomedical domain supporting the annotation of relations, and fully customizable with documents in several formats—PDF included, as well as abstracts retrieved from PubMed, Semantic Scholar, and OpenAIRE. To meet any user need, we released *MetaTron* both as an online instance and as a Docker image locally deployable.

## Background

In recent years, the exponential growth of biomedical data such as medical reports, Electronic Health Records (EHR) and physician notes posed relevant challenges in effectively and efficiently organizing, curating, managing, and reusing this data both for clinical and research purposes [1–5]. Given the textual nature of biomedical data (according to [6], the 70–80% of clinical data is text-based), extracting and reusing the knowledge in the biomedical literature can drive advances in biomedical research, enhance decision-making processes, and accelerate the discovery of drugs and diseases [3, 4, 7–9]. Consequently, Natural Language Processing (NLP) techniques have gained substantial importance as they can automate retrieval, biomedical data processing, and knowledge extraction. The research area specialized in applying NLP technique to the biomedical data is defined *BioNLP* [3]. Developing efficient and effective NLP methods is challenging as it requires the availability of large manually annotated corpora. In [10], authors address this problem and present a comparison of the most commonly employed manual annotation tools that can be used to create manually annotated corpora.

Several works studied the application of NLP technique to process biomedical data on different domains: in [11], for example, authors discuss the use of NLP techniques to extract symptoms from EHR, while [12] discuss the use of NLP to process oncology medical records. In [13], a large language model for EHR is proposed, and in [6], authors analyze NLP methods to identify advance care planning documentation in patient clinical notes.

In this context, Relation Extraction (RE) task captured considerable interest in the biomedical community as the knowledge stored in the biomedical data may contain valuable insights about the relationships between entities—e.g., protein–protein, gene–disease, drug–drug, and drug–target interactions. Furthermore, Entity Linking (EL), the task of identifying and disambiguating entity occurrences in unstructured text [14], is key to detecting the various textual representations of an entity and capturing its underlying meaning. In [4], for example, authors show how EL has numerous benefits, including better use of EHR, improved search and retrieval of biomedical resources, abbreviation disambiguation. In addition to RE and EL, another important theme in the biomedical community is Topic Recognition (TR). Detecting the topics discussed in biomedical text plays a vital role in organizing and classifying the vast amount of information available [15]. The most recent proposed techniques to perform RE [16–22], EL [23–28] and TR [29–35] on biomedical texts rely on Machine Learning (ML) models whose performances depend on the availability of large annotated corpora used in training, validation, and test. Creating sizeable and trustworthy manual annotated datasets for the biomedical domain and sub-domains is a time-consuming task requiring people with a high level of expertise [6]. In this respect, the creation of these corpora is made even more challenging by the intrinsic diversity in topics and concepts within the biomedical field; the UMLS Metathesaurus [36], for example, contains over 3.5 million unique concepts belonging to 127 different semantic types [27]. In addition, biomedical terminology is complex, and some terms may have different meanings depending on the context where they are used [37]. Some notable examples of large annotated corpora are [9, 38–45]. Furthermore, some annotated corpora targets the annotation of relationships such as [18, 46, 47]. Given the importance of the annotation task and the significant effort required by experts, several manual annotation tools have been developed explicitly for the biomedical domain [48–53]. Other tools, on the other hand, are general-purpose [54–58] and offer different features that are not aligned with the biomedical experts’ needs. The coexistence of multiple annotation tools arises from other tools offering diverse functionalities and targeting various domains of interest.

**Fig. 1:**
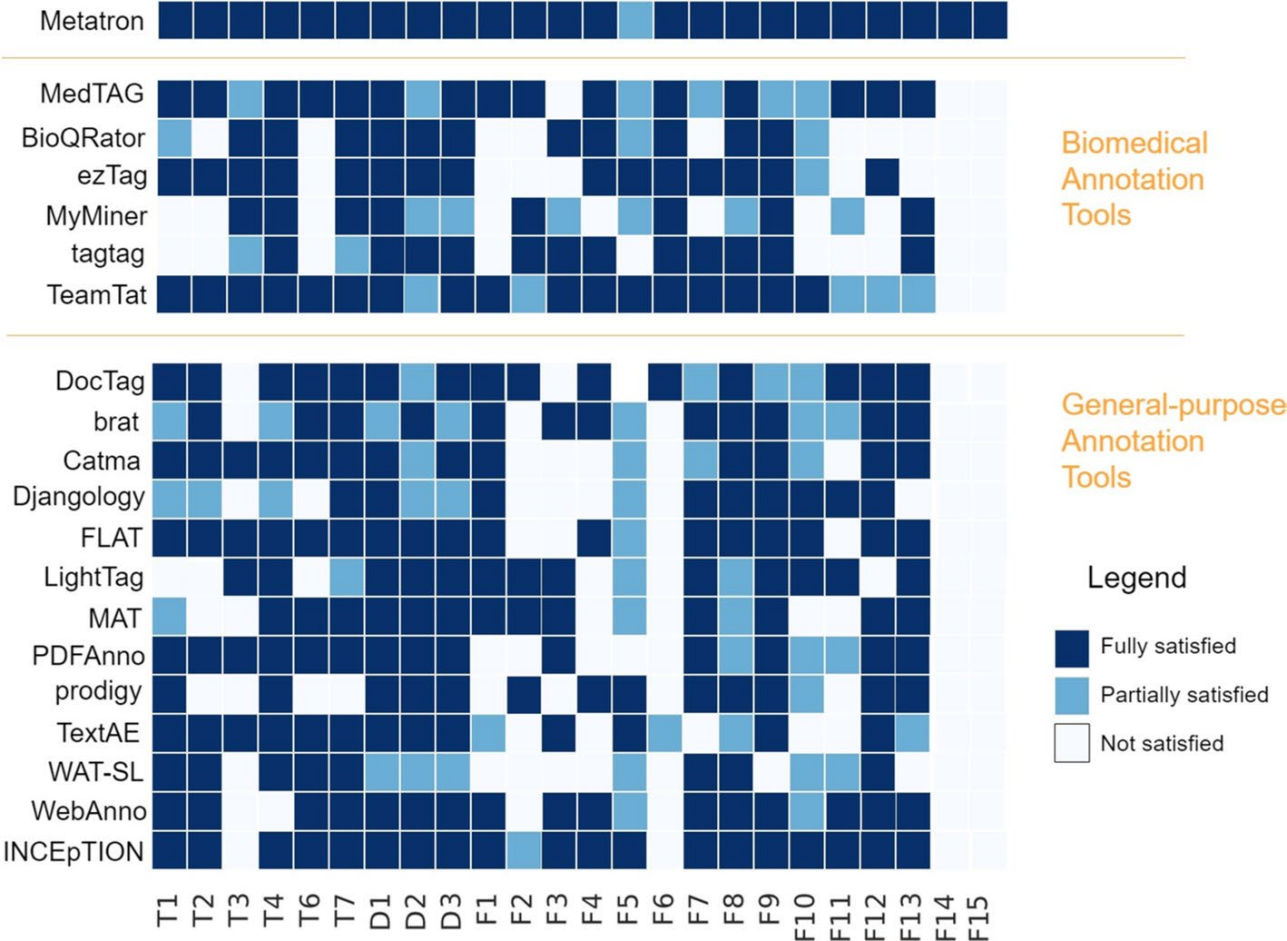
Annotation tools features overview. Each of the selected twenty annotation tools is evaluated based on 24 criteria: 7 technical (T) criteria, three criteria about input and output data formats (D), and 15 criteria concerning the functionalities (F) provided by each tool. The first 22 criteria are taken from [10], while we added the last two. Each criterion is marked in blue for each tool if the feature is fully satisfied, light blue if partially satisfied, and white if not satisfied

In [10], a review of the widest-used annotation tools is proposed, highlighting the points of strength and weakness of each. They selected 15 annotation tools according to the following criteria:

- *Availability*: the tool must be readily available. This criterion ensures that the tool is accessible via a public URL or is downloadable, independently of the user’s expertise. In this respect, the availability of source code is important not only in terms of transparency and accountability but also to guarantee further development and customization according to the user’s needs;
- *Web-based*: the tool must be a web application. With the term “web application,” we refer to a software application following the most frequently used 3-tiers architecture: a presentation layer handles user interface and interaction, an application layer implements the business logic, and a data layer permanently stores data resulting from or useful to the user interaction [59]. Web-based tools can run online. Hence, they can be accessed via a public URL or offline and, in this case, can be installed as web applications. Offline web-based tools require an installation procedure to run on the user’s personal computers or distributed servers. Web-based tools are usually more flexible and solid with the passing of time, and there is a growing emphasis on software not requiring local installations [60]; they allow multiple annotators to work collaboratively on the same document in different environments and are platform independent—e.g., other operative systems and different machines.. Conversely, according to [10], non-web-based tools are stand-alone systems or plugins running on other tools and platforms. According to two analyses carried out in [10], in the past ten years, web-based tools have been more prevalent than stand-alone tools and plugins and are the most frequently used in the annotation of biomedical corpora;
- *Installability*: offline web-based tools are needed when documents and annotations must be kept private. These tools must be installed on personal computers or distributed servers, and their installation procedure must be finished in less than two hours. The ease and speed of installation and setup are crucial factors that influence the usability of a tool for a diverse range of users with varying backgrounds. Installability is a crucial feature also highlighted by another recent survey of image annotation tools [61]; indeed, installation issues are one of the main reasons annotation tools cannot be reused in the field;
- *Workable*: the tool must be intuitive, and all the features must be comprehensive enough to be used independently of the level of expertise, relying on a well-documented set of instructions and without the help of the developers. This is a pivotal factor that directly impacts the tool’s utilization. Features that are challenging to comprehend render the examined tool impractical; see also [61] for further supporting analysis on the usability of annotation tools. Therefore, it is crucial to take into account aspects related to usability and implementation;
- *Schematic*: the tool allows for schema configuration. In this context, the tool defines elements such as labels, documents, and concepts according to the user’s needs. The tool should not be developed for a specific use-case and should not provide a fixed set of labels, concepts and rules for the annotation. Concerning this point, a tool that does not allow the user to define a schema or is designed for a specific use case will likely face challenges in being reused.

Once the tools were chosen according to the aforementioned selection criteria, the authors defined a set of 26 evaluation criteria to compare them. The evaluation criteria can be subdivided into four macro-areas: (i) *Publication*: these features concern the tools’ publications and citations; (ii) *Technical*: these features concern technical aspects of the tool, and are useful to determine the availability of the tool, and the ease of installation; (iii) *Data*: these features describe what formats the tool requires in input and output; (iv) *Functional*: criteria concerning the functionalities provided by the tools. Functional criteria describe all the feature a tool provides—i.e., document-level annotations, availability of overlapping mentions, active learning, collaborative features.

While *Functional* criteria are significant in identifying the primary distinctions among tools and deciding which tool best suits the user’s needs, *Data* criteria, on the other hand, enable users to comprehend the required data formats for each tool. Consequently, users can assess whether their data needs preprocessing. A subset of these criteria has been used also for evaluation purposes in [62] that describes an ecosystem for knowledge discovery. We revised the assessment conducted by [10], updating *LightTag*, *INCEpTION*, and *MedTAG* according to our experience with each of these tools, adding some tools released after the publication of the paper and including new features we deemed as important for the current trend in bioinformatics.

The heat map we obtained is reported in Fig. 1 with the evaluated annotation tools as rows and the tested criteria as columns. The color intensity of the cells indicates the level of adherence of a tool to each criterion. We evaluated 24 criteria, including 22 criteria (from T1 to F13 in Fig. 1) from the set of 26 criteria defined in [10] and two new criteria (F14 and F15) defined here for the first time. The first six criteria are *Technical (T)*: (T1) date of last version or commit—whether the last version has been released within the past five years; (T2) availability of source code; (T3) online availability; (T4) easiness of installation; (T6) license allowing modification and redistribution; (T7) free of charge. Three criteria concern *Data (D)*: (D1) schema format—whether the schema is configurable; (D2) input format—whether the input documents follow a standard format; (D3) output annotations format—whether the annotations are based on standard formats. Finally, fifteen criteria are *Functional (F)*: (F1) support for overlapping mentions; (F2) support for document-level annotation; (F3) support for relationship annotation; (F4) support for ontologies; (F5) support for built-in prediction and active learning; (F6) integration with PubMed; (F7) suitability for full texts; (F8) support for the partial saving of an annotation (allowing the user to continue the annotation process later); (F9) support for text highlighting; (F10) support for users and teams; (F11) support for Inter Annotator Agreement (IAA); (F12) data privacy; (F13) multilingual support; (F14) connection to ORCID; (F15) retrieval of abstracts from external repositories or services. The use of most of the evaluation criteria from [10], ensures that the evaluation analysis we conducted is as objective as possible, avoiding to bias the study towards *MetaTron* strong points. Moreover, we further validated *MetaTron* against these evaluation criteria by the means of two expert-based case studies.

As remarked in [10], we confirm that no currently available off-the-shelf tool comprehensively meets all the requirements. This is also evident from Fig. 1, where the missing features in the majority of selected annotation tools are: support for relationship annotation (F3), support for overlapping mentions (F1), support for document-level annotations (F2), connection to ORCID (F14) and the integration with external repositories and services such as PubMed to retrieve publications’ abstracts (F6, F15).

In this paper, we introduce *MetaTron*, an innovative web-based annotation tool for the biomedical literature which fulfills all the selected evaluation criteria. *MetaTron* is released both as an online instance and as a Docker image deployable on a local server relying on a quick and easy installation procedure. It is fully customizable, as users can upload documents in JSON, PDF, CSV, and TXT or retrieve and upload abstracts from PubMed, Semantic Scholar, and OpenAIRE. The support for both mention-level and document-level annotation types makes *MetaTron* suitable for several use cases. Additionally, *MetaTron* supports automatic built-in predictions.

The rest of the paper is organized as follows: in “Implementation” Section we describe *MetaTron* and its features, focusing on the annotation types *MetaTron* provides and *AutoTron* for automatic built-in predictions; in “Results” Section we describe the qualitative and quantitative analyses we conducted to evaluate *MetaTron* and compare to other annotation tools; in  “Conclusions” Section we draw some final remarks.

## Implementation

### System overview

*MetaTron* is an annotation tool designed to annotate biomedical documents. One of the key features of *MetaTron* is its support for multiple annotation types. The annotation types can be classified into *document-level annotations* and *mention-level annotation*. *Mention-level annotations* concern the annotation of specific portions of the textual document and comprize *mention, concept linking*, and *relationship* annotations. Mention annotation detects the mentions in a textual document, and each mention can be linked to one or more *concepts* from an ontology—i.e., concepts linking. In this work, we use the terms *entities* and *concepts* interchangeably; in particular, we consider a *concept* as an atomic, identifiable object that has a distinct and independent existence [63]. In *MetaTron*, as in the Semantic Web realm, a concept is identified by a URI and described by a name and a type—e.g., gene or disease—making the concept human-understandable. Relationship annotation involves identifying and marking “statements” or “facts” within a text. A statement typically consists of three components: a subject, a predicate, and an object, collectively conveying a specific meaning. It is important to note that the constituents of a statement may be explicitly mentioned in the text, or they can be implicitly understood by considering the surrounding context and their association with ontological concepts.

*Document-level annotations* pertain to considering an entire textual document as a unit. In the *MetaTron* framework, there are two types of document-level annotations: *label* and *assertion* annotations. The former involves assigning one or more labels (representing individual concepts) to the document to classify its content. The latter enables the annotation of a document with a collection of assertions, subject-predicate-object triples linked to ontological concepts. These assertions provide a high-level description of the document’s content. Treating the assertions as machine-readable triples can be incorporated into a Resource Description Framework (RDF) graph, facilitating inference and knowledge representation.

*MetaTron* offers support for ontologies by enabling users to define a collection of ontological concepts identified by an identifier, a name, a type (e.g., *gene* or *disease*) and a description. Concepts are not necessarily tied to a specific ontology: this guarantees more customizability and flexibility, allowing the user to add concepts belonging not only to widely recognized and publicly accessible ontologies but also to user-designed or not yet published ontologies or vocabularies. Concepts can be uploaded in batch or added at the moment of annotation, allowing the user to enrich the set of concepts when needed.

These features of *MetaTron* enable users to annotate a diverse range of biomedical entities and relationships, including genes, proteins, diseases, drug treatments, and their associations. By allowing this customization and flexibility, *MetaTron* can be adapted to suit various use cases and user needs.

Collaborative annotation is a significant aspect of the *MetaTron* system, particularly in the context of collectively annotating a group of documents. This collaborative feature is crucial as it facilitates users to annotate documents together, improving annotation quality and accuracy. By working collaboratively, annotators can save time and enhance the overall annotation process. Through this feature, users can view annotations made by their colleagues and identify annotations that exhibit the highest agreement. Furthermore, the members of the document collection have access to comprehensive statistics about the entire collection or specific documents. This functionality enables them to monitor the progress of annotations and visually analyze the complete set of annotations, considering various annotation types and levels of agreement.

To enhance the user experience and expedite the annotation process, *MetaTron* incorporates *AutoTron*, a feature that offers automated predictions within the system. *AutoTron* is a framework designed to automatically annotate relationships and assertions, allowing users to implement their methods as desired. Leveraging automatic annotations is pivotal in enhancing and accelerating the overall annotation workflow by offering users an initial set of annotations that can be modified. The *AutoTron* system operates as a plug-and-play mechanism, enabling users to integrate their custom automatic annotation methods seamlessly. Additionally, *MetaTron* includes two pre-built methods specifically designed for automatically annotating gene-disease associations and gene expression-cancer associations, further augmenting the automated annotation capabilities.

*MetaTron* is designed to be highly adaptable and can be customized to suit any area of interest. It offers an easy-to-use customization process, where documents can be uploaded in various formats, such as JSON, CSV, TXT, and PDF, using the integration with GROBID (GeneRation Of BIbliographic Data) [64]. GROBID is open-source software that uses machine learning techniques to extract structured data such as author names, affiliations, abstracts, publication dates, and references from scientific articles. This feature helps detect the various sections of a publication. Additionally, *MetaTron* incorporates REST APIs such as PubMed, Semantic Scholar, and OpenAIRE to enable users to upload one or more PMIDs (for PubMed) or DOIs (for Semantic Scholar and OpenAIRE) and annotate related information such as the title, abstract, authors, venue, and date of publication.

Additional features of *MetaTron* are the following: (i) an easy-to-use user interface that supports the automatic saving of the annotations; (ii) integration of keyboard shortcuts to navigate between documents and perform new annotations; (iii) support for the download of annotations in JSON, CSV, BioC/XML formats; (iv) support for the upload new annotations from JSON or CSV file; (v) support for user-defined style properties such as the colors of the mentions, the size or the line height of the textual content; (vi) multilingual support; (vii) support for annotation suggestions; (viii) IAA support, implemented through Fliess’ kappa, Cohen’s kappa and majority voting; (ix) dockerized and online instance available, (x) support for multiple ontologies, (xi) connection to ORCID, (xii) support for multiple annotation rounds.

#### Architecture

**Fig. 2:**
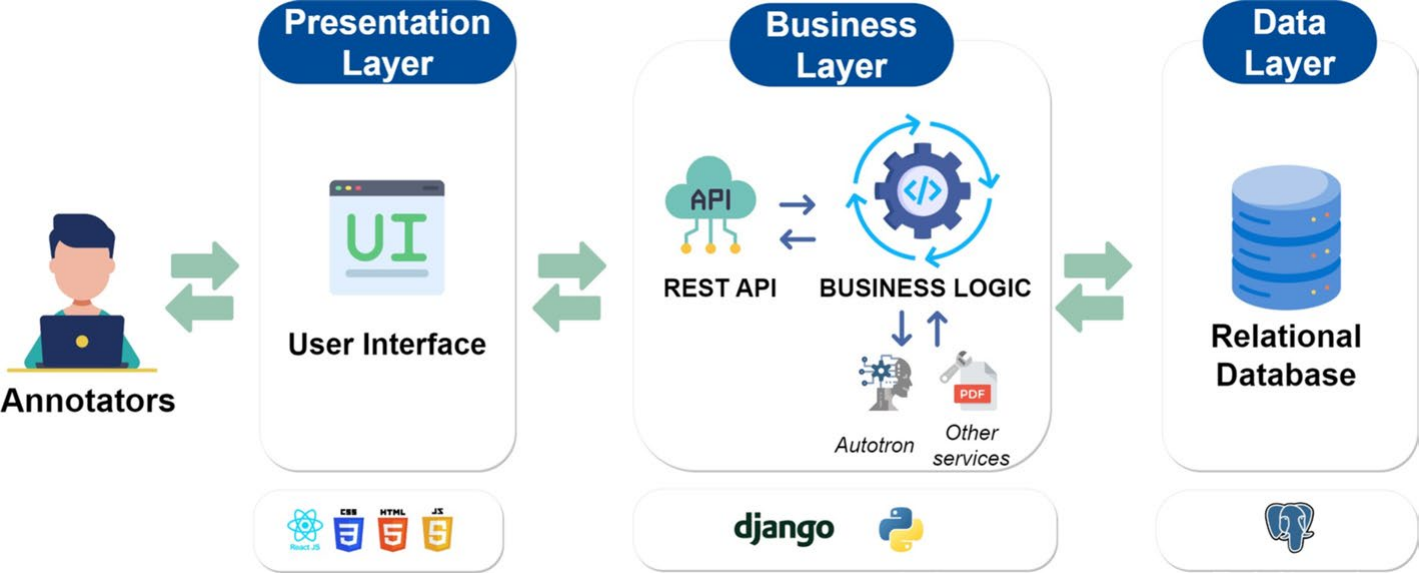
MetaTron architecture. *MetaTron* has three layers: the *Data layer* (a PostgreSQL database for data and annotations), the *Business layer* (with a REST API and services for automatic annotation and PDF parsing), and the *Presentation layer* (the user interface)

The system architecture is illustrated in Fig. 2 and can be divided into three layers: a data layer, a business layer, and a presentation layer.

The data layer is based on a PostgreSQL database that stores the annotations data, information about collections, concepts, and documents. This layer is responsible for managing the persistence and retrieval of data and ensuring data integrity.

The business layer comprises a REST API and a business logic implemented using the Django Python web framework.^1^ The REST API acts as an intermediary between the presentation layer and the data layer, while the business logic handles and processes data retrieved from the database based on the application’s needs. This layer also utilizes additional services such as *AutoTron* (presented in Sect. 2.2.2) and GROBID [64].

The presentation layer is responsible for displaying the data to the users and receiving their input. It is developed using ReactJS, HTML, CSS, and JavaScript. This layer interacts with the business logic layer through the REST API to retrieve and display the data to the user.

Overall, this architecture provides a clear separation of concerns between the different layers, improving the system’s maintainability, scalability, and modularity. Using a database, a REST API, and additional services enhances the system’s data management and processing capabilities.

#### Availability

*MetaTron* is released as an online and dockerized instance. The online instance is available at https://metatron.dei.unipd.it.^2^ An online demo and tutorial is available at: https://metatron.dei.unipd.it/demo.

It is intended to be used with scientific publications—e.g., scientific articles or publications in PubMed. To use *MetaTron*, it is necessary to sign up by providing a username, a password, and a profile that identifies the level of expertise. Once signed up, the user will be asked to specify their level of expertise. They can create new document collections and invite other collaborators to join the project.

*MetaTron* is also released as a *Docker container* which guarantees cross-platform portability, scalability, and isolation. Furthermore, the dockerized version can be utilized by users who want to upload collections of documents whose content must be kept private, in all the cases where the network is not fully operational or when users want to introduce new features (i.e., new methods for automatic relation extraction). A local installation is also required if the user works with confidential documents or in a privacy-preserving setting. The dockerized version also eases the installation of *MetaTron* in a private network setting when the documents are unavailable on the Web, but distributed collaboration amongst the annotators is required. The installation procedure is detailed in the *MetaTron* repository (https://github.com/GDAMining/metatron/), where the source code is publicly available.

### Annotation interface

**Fig. 3:**
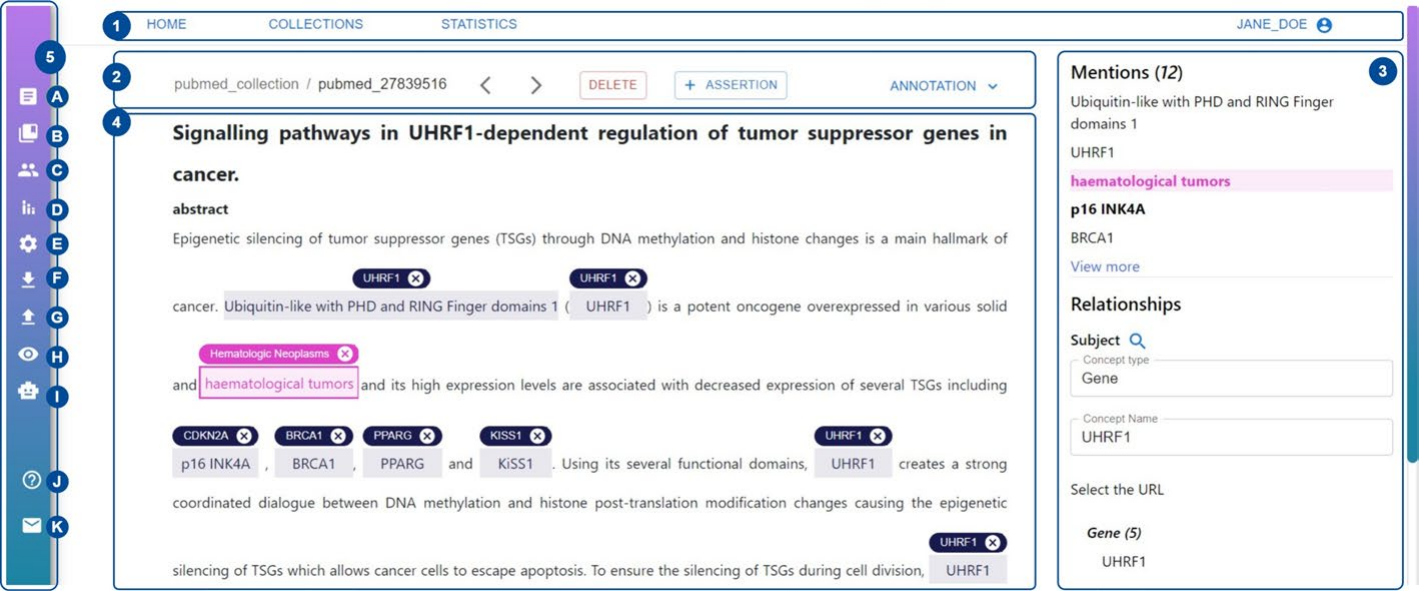
MetaTron user interface. The main annotation interface consists of five distinct blocks: in the main header (**1**), the user can navigate to other web pages and logout; in the document header (**2**), there is the main information about a document; in annotation panel (**3**) it is possible to check the annotation status; the document takes the largest part of the page to annotate (**4**); and in the vertical toolbar (**5**) it is possible to access several functions a user can perform during the annotation process

The *MetaTron* annotation interface and its features have been designed to be intuitive and facilitate and speed up the entire annotation process. *MetaTron* annotation interface is illustrated in Fig. 3 that we use as a guide to illustrate the tool’s main features. Upon successful login, the system presents the user with the most recently annotated document. At the top of the annotation interface, there is the *main header* (**1**). The *Home*, *Collections*, *Statistics* buttons can be used to navigate to home—i.e., the page illustrated in Fig. 3, collections, and statistics web pages. The user name is displayed at the top right of the page and can be used to log out. The *document header* (**2**), placed below the main header, includes the information about the current document identifier—e.g., pubmed_27839516—and the related collection name—e.g., pubmed_collection. Two arrow buttons allow the user to navigate between the documents of the collection; it is worth noting that it is also possible to navigate from one document to another through a custom shortcut designed to allow the user to change documents relying only on the keyboard. The *Delete* button deletes the annotations for the current document, while the *Assertion* button creates a new assertion as a document-level annotation.

By clicking on the *Annotation* button, a drop-down menu appears, enabling the user to choose one or more annotation types. For each selected annotation type, the list of annotations is then displayed on the right-hand side of the annotation interface, in the *annotation panel* (3). The user can view, add, modify, or delete their annotations using this panel. The largest portion of the page is taken by the textual document (4) the user annotates. The *vertical toolbar* (5) provides a set of functionalities illustrated in Fig. 3 from (A) to (K), that can be accessed directly from the main annotation interface improving, and speeding up the annotation procedure, and minimizing the number of actions to be performed. In (A), the list of documents of the collections is shown. The user can filter documents based on whether they contain at least one annotation and search for a specific document to annotate using its ID. In (B), the user can view a list of document collections available for annotation. Each collection is represented by a button displaying the collection name and the percentage of annotated documents. The button color varies based on the percentage of documents that have been annotated—e.g., green color is used when the user annotated more than 80% of documents, while red color is used when the user annotated less than 20% of documents. By clicking on a collection, it is possible to start annotating its documents. In (C), the users who annotated the current document are listed. It is possible to load the annotation of one of the users in the list by clicking on the associated username. (D) allows the user to open two tables containing *personal* and *global* annotations statistics overview for the current document; the former concerns the annotations of the current user, while the latter those of all the annotators. Each table contains the number of mentions, associations, mentions-concepts, relationships, assertions, and labels annotated. In the *global* overview, the agreement computed with the Fleiss’ kappa measure is provided. (E) allows the user to customize *MetaTron*. It is possible to: hide or display specific sections of the document, increase or decrease the font size and the line height, and set the color associated with each concept type. These settings have been defined to facilitate and speed up the annotation workflow and improve document readability. (F) enables to download of the annotations. The user has to specify the file format—e.g., JSON, CSV, BioC, the annotation type—e.g., mentions, concepts, relationships, and the annotator username, choosing between all the users who annotated that document. (G) opens an upload panel where only the user who created the collection can upload new lists of documents and ontological concepts. Documents can be uploaded in several formats: CSV, TXT, JSON, and PDF. The user can search for a specific publication in PubMed (by providing the related PMID), in Semantic Scholar, and OpenAIRE (by providing the related DOI): *MetaTron* takes advantage of their REST APIs to retrieve the title, the abstract, the authors, the date of publication, and the venue information. The user can upload a new set of annotations in CSV or JSON format that will be automatically loaded in *MetaTron*. In (H), the user can hide the ontological concepts associated with the mentions and visualize only the annotated mentions increasing the document’s readability. In (I), it is possible to rely on *AutoTron* to annotate the current document automatically for specific cases. (J) and (K) open new tabs with the *MetaTron* instructions, and the credits respectively.

#### Manual annotation

*Mention annotation* Mentions are textual spans that can be linked to one or more ontological concepts. In *MetaTron*, a mention can either consist of one or more consecutive tokens (or words), where a token is a sequence of characters between two spaces or a substring of one or more contiguous tokens. In this case, the first or last character of the mention does not necessarily coincide with the character that follows or precedes a space. It is possible to select a mention that consists of two or more consecutive tokens by clicking on the first and the last words, respectively: all the words comprised between the two selected will be part of the new mention. To create a mention containing a single token, it is possible to double-click on the desired token. To annotate a substring, hence selecting a part of the token (or two or more consecutive tokens), it is possible to drag and drop the mouse from the first to the last characters of the substring. *MetaTron* allows for the selection of overlapping mentions, meaning that a piece of text already included in a mention can be selected.

This implementation is based on our direct experience with other annotation tools, which has allowed us to assess the pros and cons of various possible implementations. We have decided to implement the annotation of mentions both through drag and drop and by clicking on individual words to allow the user to annotate both specific textual substrings and mentions of two or more words quickly, streamlining and expediting the workflow.

When the textual content of a mention occurs more than once in the textual document, it is possible to annotate all the mentions simultaneously. When a mention is annotated, a new modal will appear if its content occurs more than once, and the user can decide to annotate the mentions altogether.

**Fig. 4:**
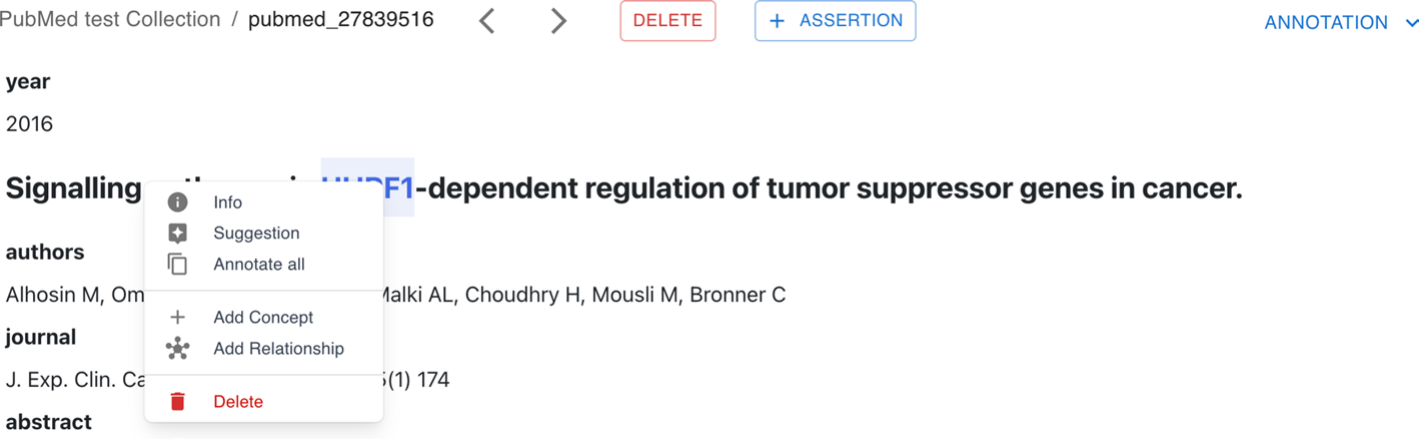
MetaTron mention panel. The mention panel opens when the user right-clicks on the desired mention. It allows the user to get more information about the mention, receive suggestions about the concepts to link, add new concepts or relationships, annotate similar mentions, and delete the mention

It is possible to access the *mention panel* illustrated in Fig. 4 by performing a right-click on a mention. This panel includes all possible actions and annotations related to the selected mention. From this panel, users can get information about the mention—e.g., the date of annotation and the number of annotators who annotated that mention for that document, receive some suggestions about the concepts to link to the mention, perform new annotations—i.e., add a new concept or a new relationship, and delete the mention. The option *Annotate all* finds all the occurrences of a mention in the document and annotates them simultaneously.

*Concepts linking* To link a new ontological concept to a mention, the user can open the mention panel of a mention and select the *Add Concept* option. A new modal will appear, allowing the user to select a concept. Each collection of documents has a list of user-defined ontological concepts, not necessarily tied to a single ontology. From the modal, the user can explore this set of concepts, filter them according to their type, name, and identifier, and view the associated description. If the list of ontological concepts of the collection is large, to aid the user in selecting a concept, *MetaTron* provides auto-completion facilities to find the desired concept easily.

The incorporation of concept annotations in our system mirrors the approach employed by various tools, such as *brat* and *INCEpTION*. Nevertheless, we observed that our implementation is characterized by its intuitiveness. By positioning the concept above the corresponding mention and employing distinct colors based on the type, users can readily discern between different concepts, thereby augmenting the immediacy of the annotation experience.

Alternatively, if the concept is unavailable in the provided list, the user can define a new concept that will be automatically added to the collection’s concepts list. In this case, the user must define the concept’s type, name, URI (or ID), and an optional description.

**Fig. 5:**
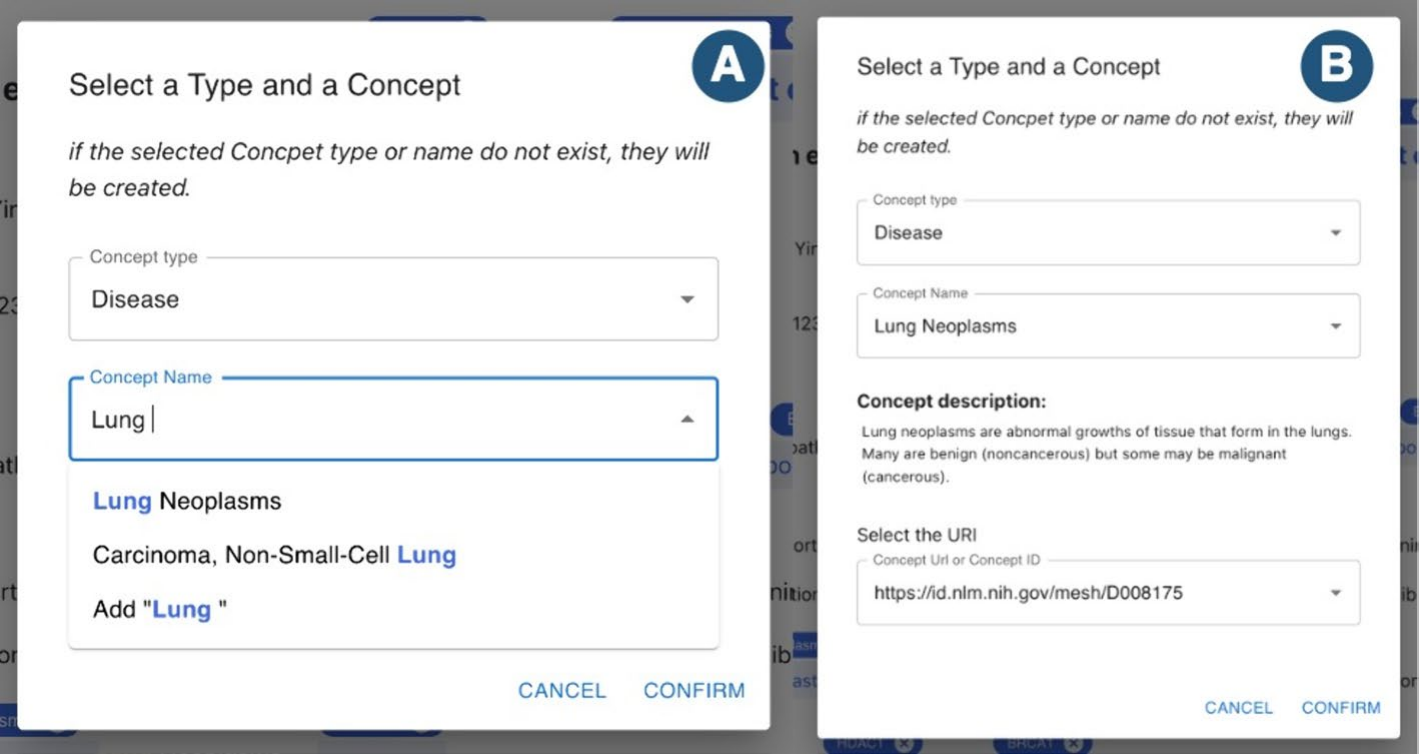
MetaTron concept selection modal. The concept selection modal allows the user to select a concept to link to a mention. The user can filter the concepts according to the concept type and use auto-completion facilities to filter further the list of concepts (**A**). Once a concept is selected, its description will automatically appear in the modal (**B**)

In Fig. 5, we can see how to link a concept to a mention. The user can filter the concepts to choose from, specifying the concept type—i.e., *disease* in the figure. By typing the first letters of the desired concepts, the available options are automatically shown (A in Fig. 5). The required information typically consists of the concept type and the name to select a concept. The user is required to select the ID of the concept only if two or more concepts share the same name but with different URIs. Once a concept is selected, the related description will automatically appear (B in Fig. 5). If no option is shown, the concept is not on the list, and it is possible to add a new concept.

If a mention is linked to one or more concepts, by clicking on the *Annotate all* option of the mention panel, it is possible to locate all the instances of that mention in the document and associate them with the same set of concepts simultaneously.

Each mention can have more than one linked concept. The concepts linked to a mention are displayed above the mention; they are clickable so that the user can be provided with the information about the concept—i.e., the URI, the name, the description, and the type. In the annotation interface, each concept can have a different color depending on its type: in Fig. 3, for example, concepts of type *Disease* and the associated mentions are highlighted in pink, while *Gene* type concepts in dark blue.

**Fig. 6:**
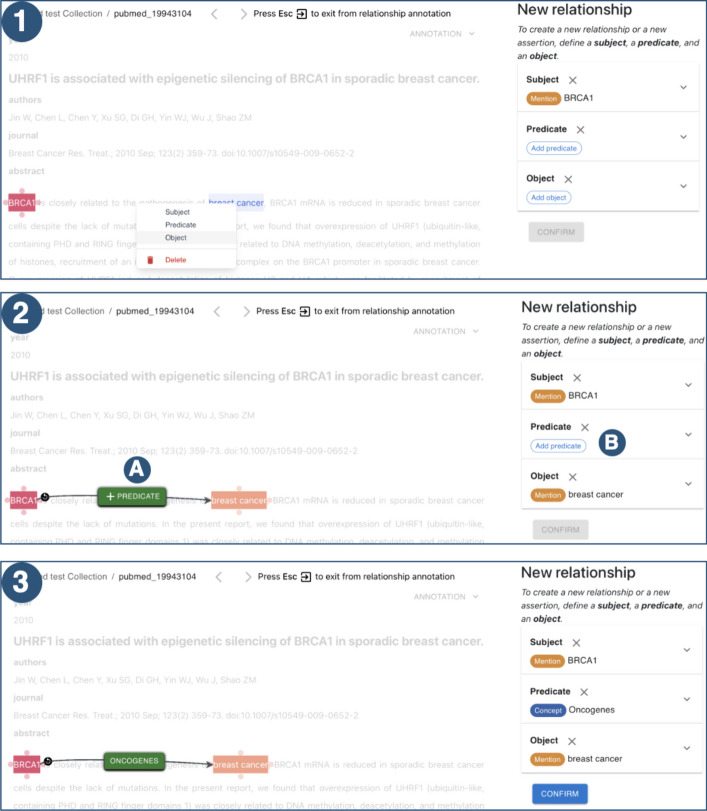
Relationship annotation. When the user annotates a new relationship, the document’s content is blurred except for the mentions highlighted with different colors, which can be selected as subject, predicate, and object, respectively. The three steps to create a relationship are shown in the example examined. First, the object mention is selected from the menu (**1**); then, it is selected a predicate (**2**): by clicking on **A**, it is possible to manually type the predicate of the relationship, while by clicking on **B** it is possible to select a concept. In (**3**), the final relationship is represented: it comprises two mentions—the subject and the object and an ontological concept—the predicate. Each relationship element has a different color to make each element easily recognizable. The panel on the right allows the user to update and save the relationship

*Relationship annotation* A relationship comprizes three primary components: a *subject*, a *predicate*, and an *object*; the relationship always starts from the subject and ends with the object. Each relationship element can be represented by either an ontological concept or a textual mention (with or without linked concepts). At least one of the three components of a relationship must be a mention.^3^ A new relationship can be added through the *Add Relationship* option of the mention panel of a mention. By default, this mention will be the subject of the relationship. This action automatically shows a *relationship panel* that provides a comprehensive overview of the relationship and its components. All the other mentions composing the relationship (if any) can be added by clicking on each mention, or by right-clicking on the desired mention, it is possible to select its role. It is worth noting that it is always possible to change the role of a mention—e.g., a mention which was the subject can become the object. From the relationship panel, it is possible to select the concepts of the relationship by clicking on the *Add predicate*, *Add subject*, or *Add object* buttons. In Fig. 6, the creation of a new relationship is illustrated. The relationship comprises two mentions (the subject BRCA1 and the object breast cancer) and an ontological concept (the predicate Oncogenes). In **1**, the object mention is selected by declaring its role from the menu. In **2**, the predicate is selected. There are two ways to select a predicate: from **A**, it is possible to input a string manually that represents the predicate, while from **B**, it is possible to select a predicate concept. In **3**, the created relationship is shown. In the textual document, each relationship component has a different color depending on its role: the subject is highlighted in red, the predicate in green, and the object in orange. The three components are linked via arrows whose positions can be changed by the user to facilitate the document readability—i.e., each mention is surrounded by four points that determine the points where the arrows can start or end.

On the right, the *relationship panel*, for each relationship component, shows the type of the component—e.g., mention or concept, and its role—e.g., subject, predicate, object. The annotations panel provides an overview of all the relationships the user annotates; each relationship can be viewed, edited, and deleted directly from the interface.

In this implementation, each relationship component is manually chosen, whether a mention selected in the text or a concept chosen relying on the right panel. This approach, even though it may require more steps than previous annotations, allows the users to designate which element within the relationship should be identified as a mention and which as a concept. Unlike *brat* and *INCEpTION* that enable the annotation of relationships, *MetaTron* does not necessitate the subject and object to be mentions within the textual document. Relationships may span multiple sentences; at least one among subject, predicate and object must be a mention, while the remaining components can be concepts.

**Fig. 7:**
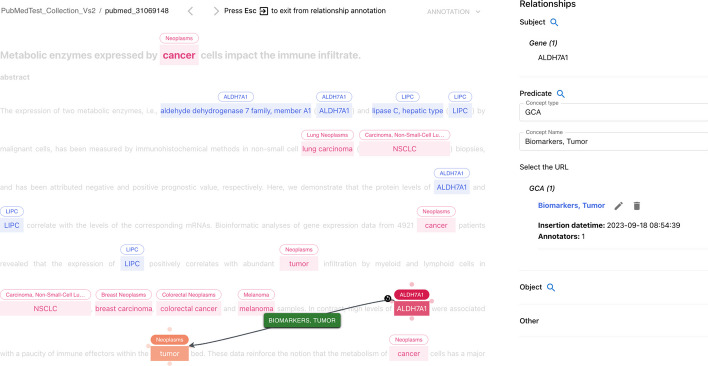
Relationships list. Overview of the relationships annotated by the user. The list is subdivided into subject, predicate, and object, each into concept types. It is also possible to filter the concepts composing the relationships according to the type and the name, taking advantage of auto-completion facilities

In Fig. 7, we can see the relationships list. It is subdivided into three categories: subject, predicate, and object; each category is further subdivided into concept types. This layout provides the user with an overview of the different concept types characterizing subjects, predicates, and objects of the annotated relationships.

**Fig. 8:**
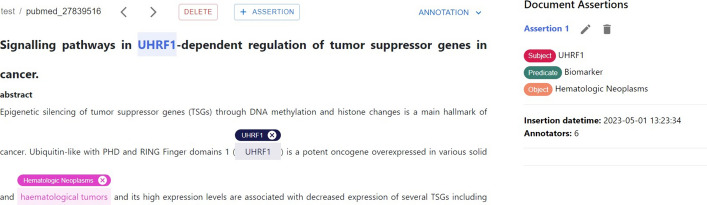
Assertion details. The annotation panel contains an overview of the annotated assertions. For each assertion, the subject, the predicate, and the object concepts are provided; each assertion can be edited or deleted via the two buttons placed near the *Assertion 1* title. The date of annotation and the number of annotators are shown below the assertion

*Assertions annotation* Like relationships, assertions consist of a subject, predicate, and object, each represented by an ontological concept unlinked to any mention in the document. To add a new assertion (see Fig. 8), a dedicated button in the document header opens an *assertion panel* similar to the one provided for relationships. Users can create a new assertion by specifying the types, names, and URIs of the subject, predicate, and object concepts. The annotated assertions can be viewed, edited, and removed via the annotation panel by selecting the assertion annotation type; the annotation panel contains the assertions the user created. For each assertion, the subject, the predicate, and the object concepts are provided with information, including the date and number of annotators. Furthermore, each assertion can be viewed, edited, or deleted. As far as our current knowledge extends, *MetaTron* stands out as the initial tool to introduce the creation of document-level assertions. Therefore, we have opted to maintain a close resemblance between assertion annotations and relationship annotations. This decision aims to facilitate a seamless user experience, enabling users to bypass the need to acquaint themselves with a novel annotation methodology and expedite the overall annotation workflow.

**Fig. 9:**
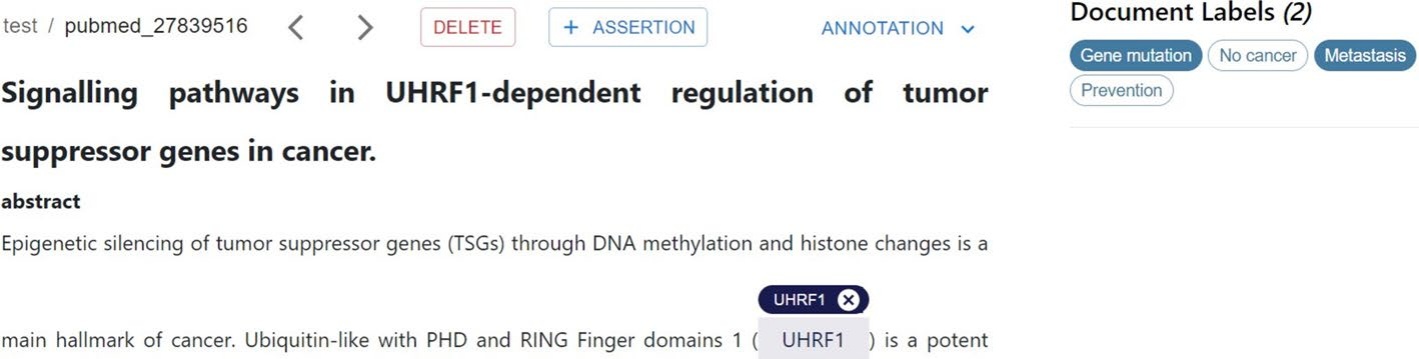
Labels annotation. The annotation panel contains the labels to assign to the document. Each label is a button that can be selected or deselected. All the selected labels have a colored background; the others have a transparent background

*Labels annotation* Labels allow the user to classify the document. Each collection has its own set of labels specified at its creation. To add one or more labels to the document, the user must open the annotation panel and select the labels annotation type. Each label is a button that can be selected or selected by a click. In Fig. 9, the annotation panel shows an example of the labels that can be associated with the displayed document for the selected collection. The selected labels have a light blue background.

#### AutoTron: automatic annotations

**Fig. 10:**
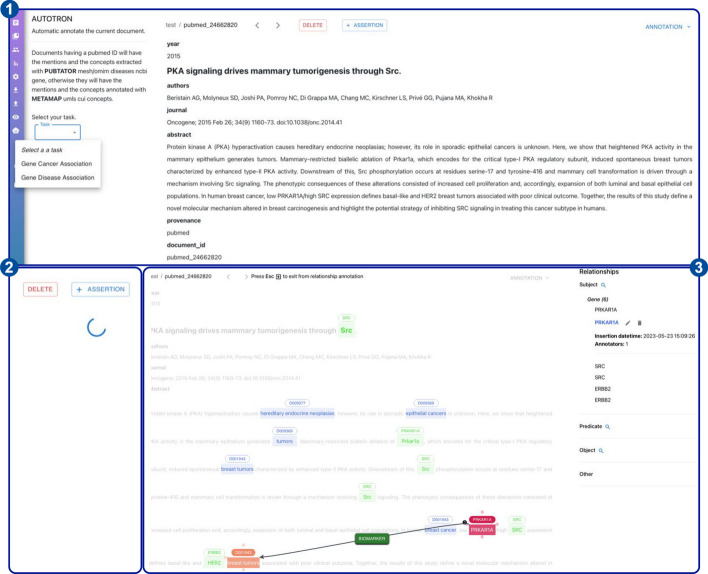
AutoTron workflow. Overview of the workflow to extract GCAs from PubMed article 24662820. In (1), users select the task (i.e., GCA). Then, once the annotation process starts, a loading icon indicates the process is underway (2). Finally, the generated annotations are displayed to users in (3)

*AutoTron* represents the automatic annotation component of *MetaTron*. As an automatic component, *AutoTron* can be implemented by the user at will. The only requirement lies on the I/O structure, where specific I/O data are required to integrate the component within *MetaTron*. Below, we first describe the general architecture of the *AutoTron* component and then present two different implementations used in two annotation tasks: document-level Gene-Disease Association (GDA) extraction and sentence-level Gene expression-Cancer Association (GCA) extraction. Both implementations are currently available in *MetaTron* and can be used by the user on the corresponding annotation tasks. Figure 10 shows the *AutoTron* workflow to extract GCAs from a sample PubMed article (i.e., PubMed id: 24662820). The workflow involves three steps. First, users select the desired task (1), which in this case is GCA. Once the user clicks the *annotate* button, a loading icon indicates that the automatic annotation process is underway (2). Finally, the annotations generated by *AutoTron* are displayed to the user (3).

*Architecture* *AutoTron* consists of two main components and specific I/O data requirements. The main components are EL and RE. EL assigns unique meanings to entities mentioned within text [65, 66], whereas RE identifies and extract relations between linked entities mentioned in text [67, 68]. The EL and RE modules are containers where different methods can be plugged in/out, but which must adhere to specific I/O data. In this regard, the I/O data consists of specific fields. In input, *AutoTron* requires a field containing the text to annotate. In the output, *AutoTron* provides, for each extracted relationship or assertion, the subject, predicate, and object concept IDs, names, and types, as well as the corresponding mention positions within the text (if any). Together, these fields allow *MetaTron* to seamlessly integrate any implementation of the *AutoTron* component. In other words, *AutoTron* represents a framework for automatically annotating relationships and assertions that users can implement at will.

*Entity linking* We consider different EL systems depending on the input text. When the input text comes from PubMed, we use the PubTator system [69–71]. PubTator provides automated annotations from state-of-the-art text mining systems for genes/proteins, genetic variants, diseases, chemicals, species, and cell lines. In particular, PubTator normalizes annotated genes to NCBI Gene [72] identifiers and annotated diseases to MeSH [73] identifiers. When the input text comes from sources different than PubMed, we use the MetaMapLite system [74], a near real-time EL tool that identifies UMLS [36] concepts within the biomedical text. MetaMapLite returns, among other information, the CUI, the preferred term, and the location in the text of the identified UMLS concepts.

The text annotated by EL systems is then passed to RE methods to perform GDA/GCA extraction.

*GDA extraction* The discovery of GDAs is one of the most pressing challenges to advance precision medicine and drug discovery [75]. Therefore, the automatic extraction and curation of GDAs is pivotal to advancing precision medicine and providing knowledge to assist disease diagnostics, drug discovery, and therapeutic decision-making. To this end, we use a document-level RE method that adopts Multi-Instance Learning (MIL) to extract GDA assertions from text [76, 77]. Under MIL, text sentences are divided into bags based on pairs of concepts, and the prediction of relations (i.e., predicates) occurs at the bag level. The use of MIL well suits the assertion annotation task, where subject, predicate, and object are not associated with mentions. As the underlying ML model, the RE method exploits Piecewise Convolutional Neural Network (PCNN) model [21]. PCNN first encodes sentences using a CNN and then applies a piecewise max pooling operation. This operation divides each sentence into three segments based on the positions of the two given entities and returns the maximum value in each segment instead of a single maximum value over the entire sentence. For MIL, the RE method performs average-based aggregation. This aggregation strategy assumes that all sentences within the same bag contribute equally to the bag-level representation. In other words, the bag representation is the average of all its sentence representations.

We trained the method on the TBGA dataset [78], a large-scale, semi-automatically annotated dataset for GDA extraction. TBGA contains over 200,000 instances and 100,000 bags, divided into four GDA types: Therapeutic, Biomarker, Genomic Alterations, and NA. Once trained, the RE method is deployed within *AutoTron*.

*GCA extraction* Cancer prevention is one of the century’s most pressing challenges that public health needs to face. In the last few years, the rise of microarray and next-generation sequencing technologies triggered the generation of large amounts of raw experimental data about gene expression-cancer interactions. These raw data require investigation, processing, and validation by experts to be used to guide diagnosis, assess prognosis, or predict therapy response [75, 79]. The outcomes of experts’ analyses are (often) described in scientific publications in the form of GCAs. However, manual knowledge extraction requires high economic and time costs [80–82]. Thus, it is of paramount importance to assist manual GCA extraction through the use of automated methods. In this regard, we use a sentence-level RE method that combines the outcomes of different models to obtain the corresponding GCA. Specifically, the method combines three ML models, each of which predicts a specific aspect associated with GCAs. The considered aspects are the Change of Gene Expression (CGE), the Change of Cancer Status (CCS), and the Gene-Cancer Interaction (GCI). Once predicted, the different aspects are combined—following a set of inference rules defined in [47, 83]—to infer the role the given gene has on the specific cancer disease. All the ML models adopt SciBERT [84], a pre-trained language model based on BERT [85]. SciBERT addresses the lack of high-quality, large-scale labeled scientific data by pretraining on scientific papers from Semantic Scholar [86]. On top of it, a linear layer takes SciBERT pooled output. Predictions are scores in [0, 1]; the higher the score for an aspect value, the more the model believes the sentence expresses that particular (aspect) value.

The method has been used to build a large-scale Knowledge Base (KB) on GCAs [87, 88]. In this work, we deploy the method as is within *AutoTron*.

### Collections and customization

**Fig. 11:**
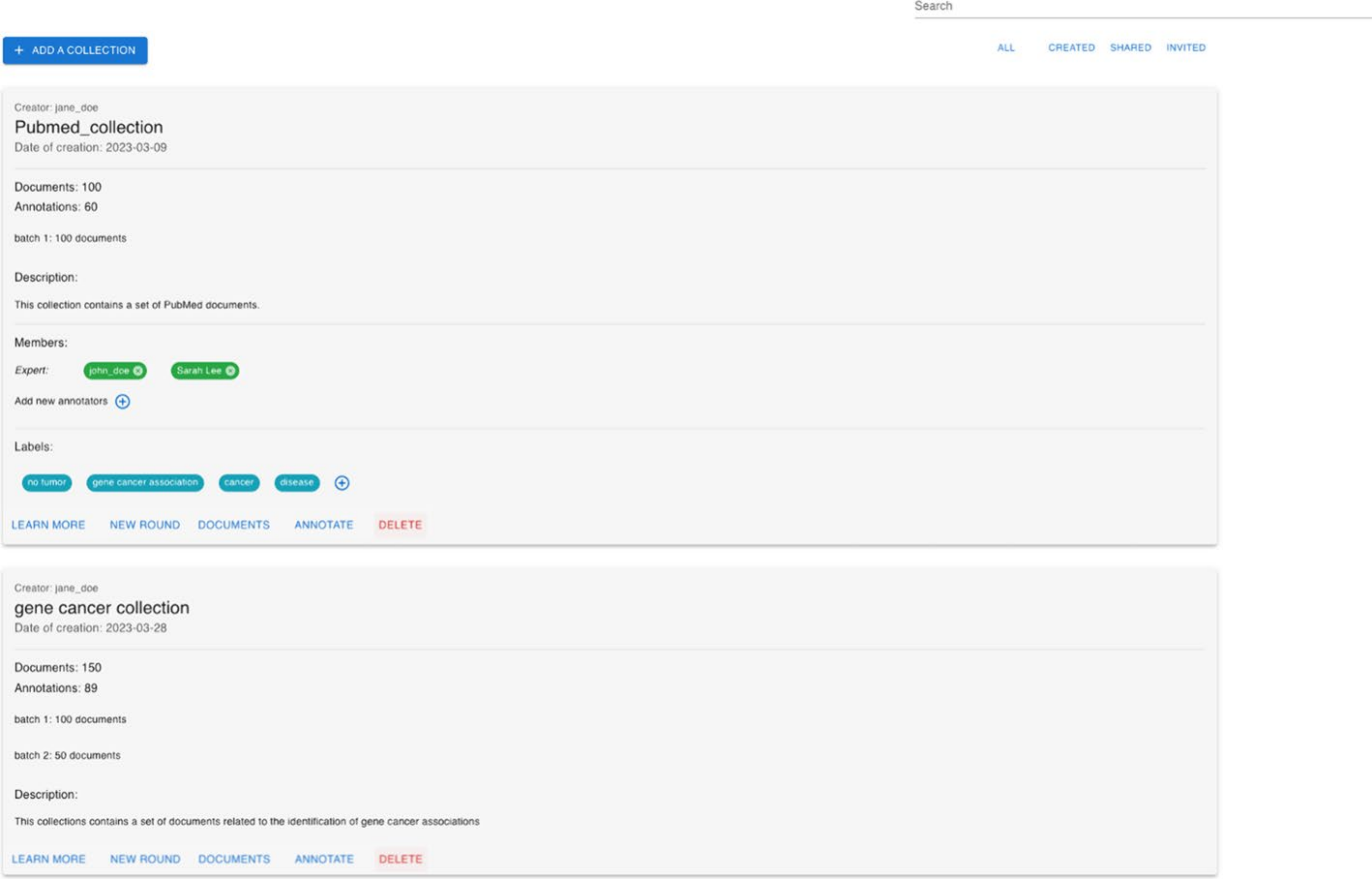
MetaTron collections interface. Overview of the main components of the collection page. The user can search for a specific collection or filter the collections list at the top-right. The *Add collection* button allows the user to create a new collection. The collections list takes up the largest part of the page. Each collection includes information that the collection’s creator can edit

Collections are sets of documents that one or more users can annotate. The collection web page is illustrated in Fig. 11, and is accessible through the annotation interface by clicking the *Collections* button.

At the top of the collection page is a text field that allows users to search for a specific collection by its name. Additionally, the four buttons below enable users to filter the collections. The default filter is *All*, which provides users with a list of all the collections they can annotate. *Created* button shows collections created by the user, while *Shared* displays collections that the user can annotate and be created by another team member. Lastly, the *Invited* button shows collections the user has been asked to join as an annotator. A new collection can be created via the *Add Collections* button. Finally, in the remaining part of the page, the user’s collections (either the entire or filtered list) are listed. For each collection, the following information is provided in this order: the creator, the collection’s name, the date of creation, the number of documents, the number of documents annotated by at least one user, and the description; by clicking on *Learn More* button, located below the collection, the information about the annotators of the collection, and the list of labels are loaded. If the user is also the collection’s creator, they can add or remove one or more annotators and add new labels. The *New Round* button allows the user to create a new annotation round. Depending on the annotation task, the users can perform one or more rounds of annotation; hence, they annotate the collection documents several times. The collection’s creator can also decide on the annotators of each round so that different sets of annotators can contribute to different rounds. This option automatically duplicates the annotations of the last round and makes them available in a new collection. The annotators who access this new collection are provided with all the annotations they performed in the last annotations round for that collection. Encapsulating each round on a separate collection of documents and annotations allows each round to be independent of all the other rounds and facilitates the annotators’ work. The *Documents* button redirects the user to the collection’s documents web page containing the list of documents of the collection together with its annotations—a more detailed description of this table is provided in Sect. 2.4. The *Annotate* button allows the user to annotate that collection: the user will be automatically redirected to the annotation interface and provided with the last annotated document of the collection (if any, otherwise with the first document available). Only the creator is provided with the *Delete* button, which allows for the deletion of the entire collection and the related annotations.

**Fig. 12:**
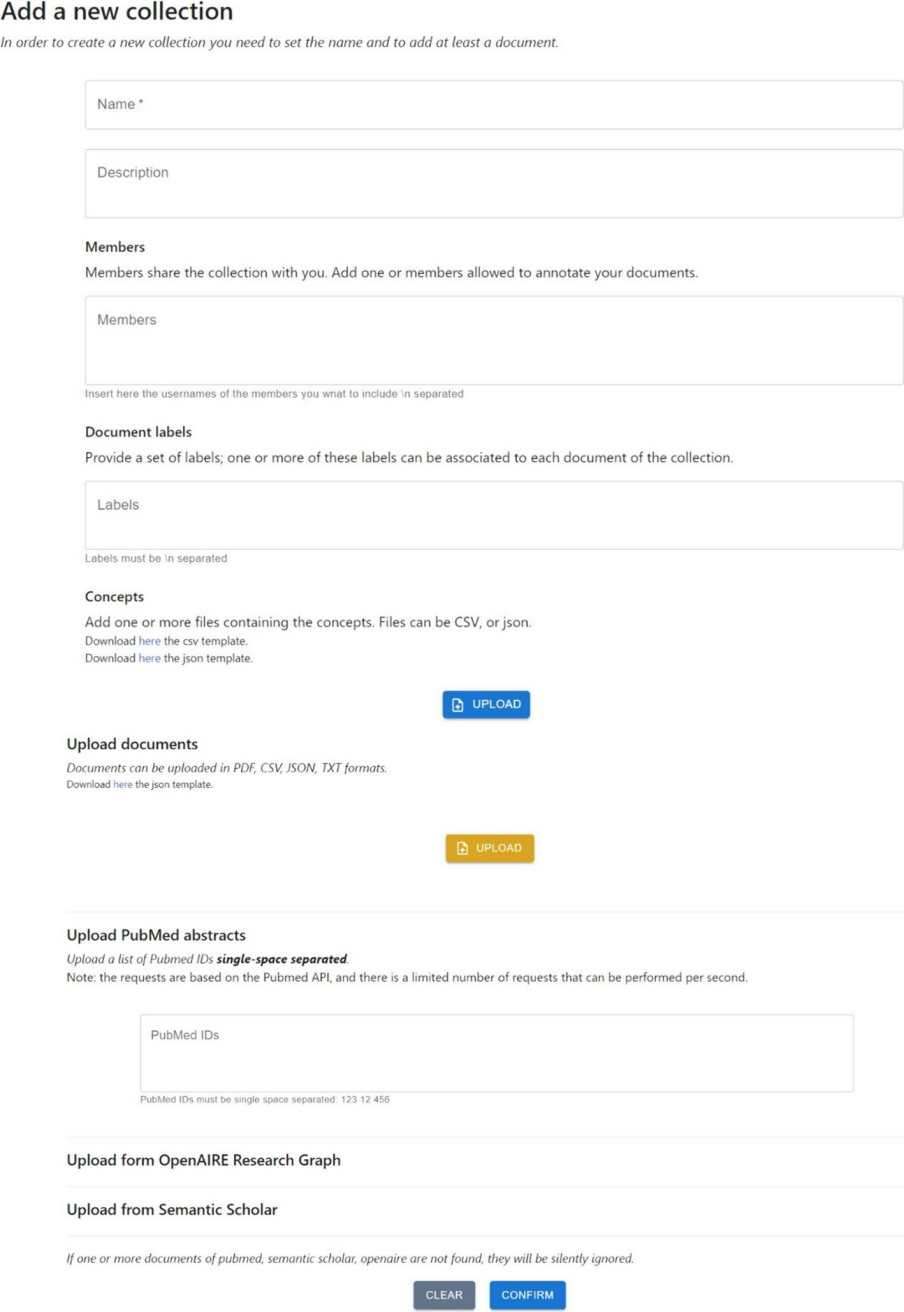
MetaTron new collection form. This figure shows the information a user must provide to create a new collection of documents. The form must provide the collection’s name and description, a list of concepts, labels, and documents

In Fig. 12, the form to add a new collection is illustrated. A user must provide the collection’s name and description to create a new collection. Then, the user is asked to provide a list of members authorized to annotate the new collection. This is not mandatory since a user may decide to work independently on a collection. The user is asked to add a list of labels necessary to perform label annotation. Also, in this case, adding a list of labels is not mandatory at the moment of collection creation. The creator can edit the collection’s annotators and labels at any moment. Uploading a set of ontological concepts is highly recommended to perform concept linking, relationship annotation, and assertion annotation. Ontological concepts must be uploaded in CSV or JSON files, and for each concept, it is mandatory to provide the URI (or ID), the name, and the type; a description is not mandatory but recommended. Since the files introducing new concepts must follow predefined structures, we provided two downloadable templates (one for the CSV and one for the JSON formats). It is worth noting that the set of provided ontological concepts is not tied to a specific ontology, allowing the user to fully customize the collection’s configuration with concepts belonging to different ontologies, which may also not be publicly available. The insertion of new concepts is always possible at any moment.

A collection must contain at least one document. The creator can upload one or more files in the following formats: JSON, CSV, TXT. The keys in JSON files and the CSVs’ headers will also be available for annotation. *MetaTron* supports uploading PDF files automatically parsed by GROBID.

To retrieve information about articles having a PMID, *MetaTron* relies on the PubMed REST API to obtain the article’s title, abstract, date of publication, authors, and venue. For publications with a DOI, *MetaTron* utilizes the REST APIs of OpenAIRE and Semantic Scholar to extract the same information. The information obtained from the REST APIs is extracted, processed, and presented to the collection users as documents that can be annotated.

By default, *MetaTron* provides the user with the entire document to annotate. However, the user can select specific parts of the document using the *settings* option in the vertical toolbar of the annotation interface.

### Collaborative features

**Fig. 13:**
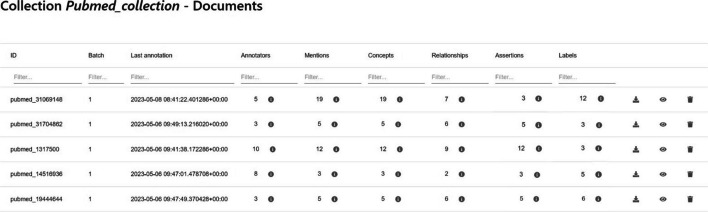
MetaTron documents collection. Overview of the documents web page related to a collection of interest. Each document is the row of a table; each row contains the information about a document, the annotators, and the total count of annotations performed for each annotation type. For each annotation type, it is possible to see an overview of the related annotations. The last column contains buttons allowing the user to download, visualize and delete the document

**Fig. 14:**
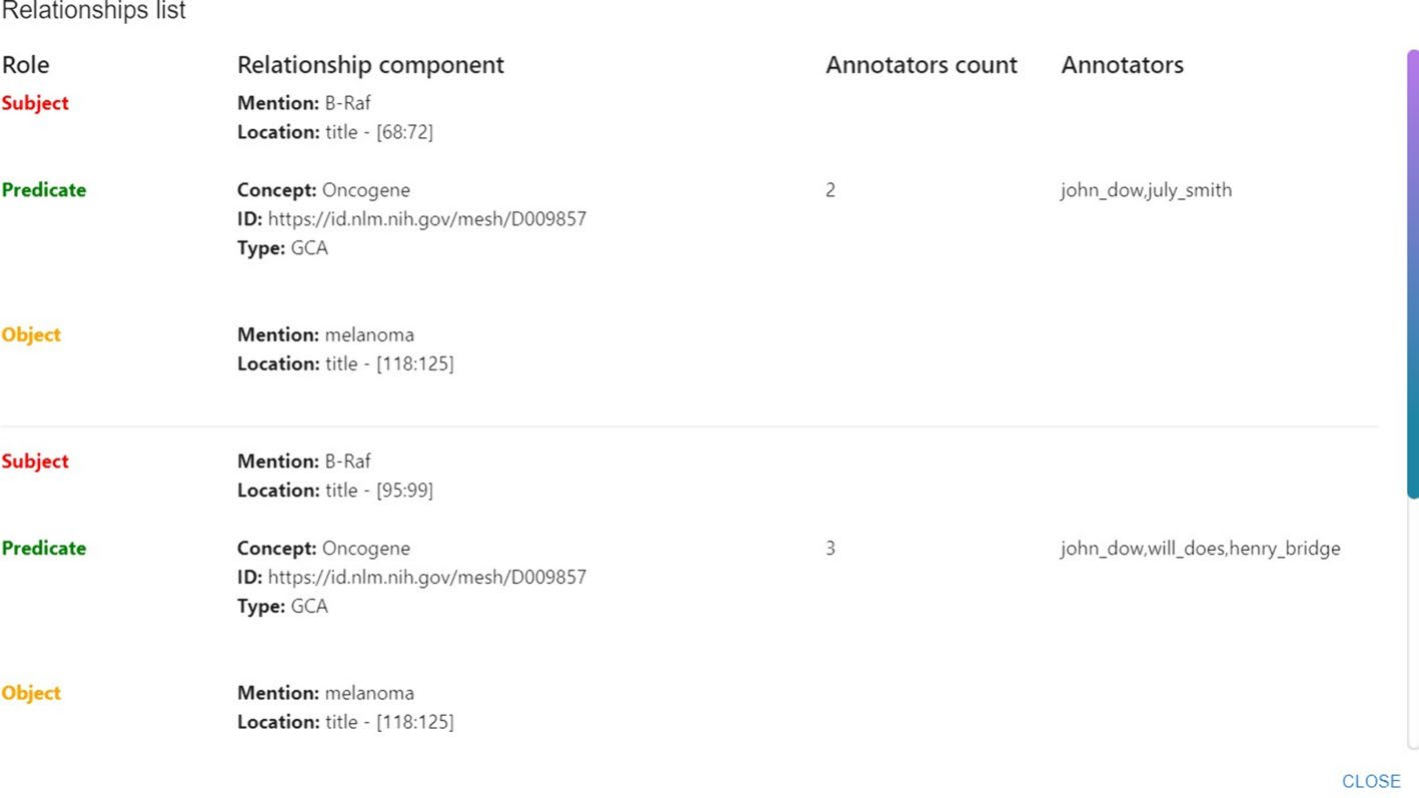
MetaTron relationships overview modal. The modal shows the subject, predicate, and object elements for each relationship annotated in the document. The mention text and the location in the text are reported; for each concept, the type, name, and URI are reported. Finally, the modal shows the number of annotators and their usernames

**Fig. 15:**
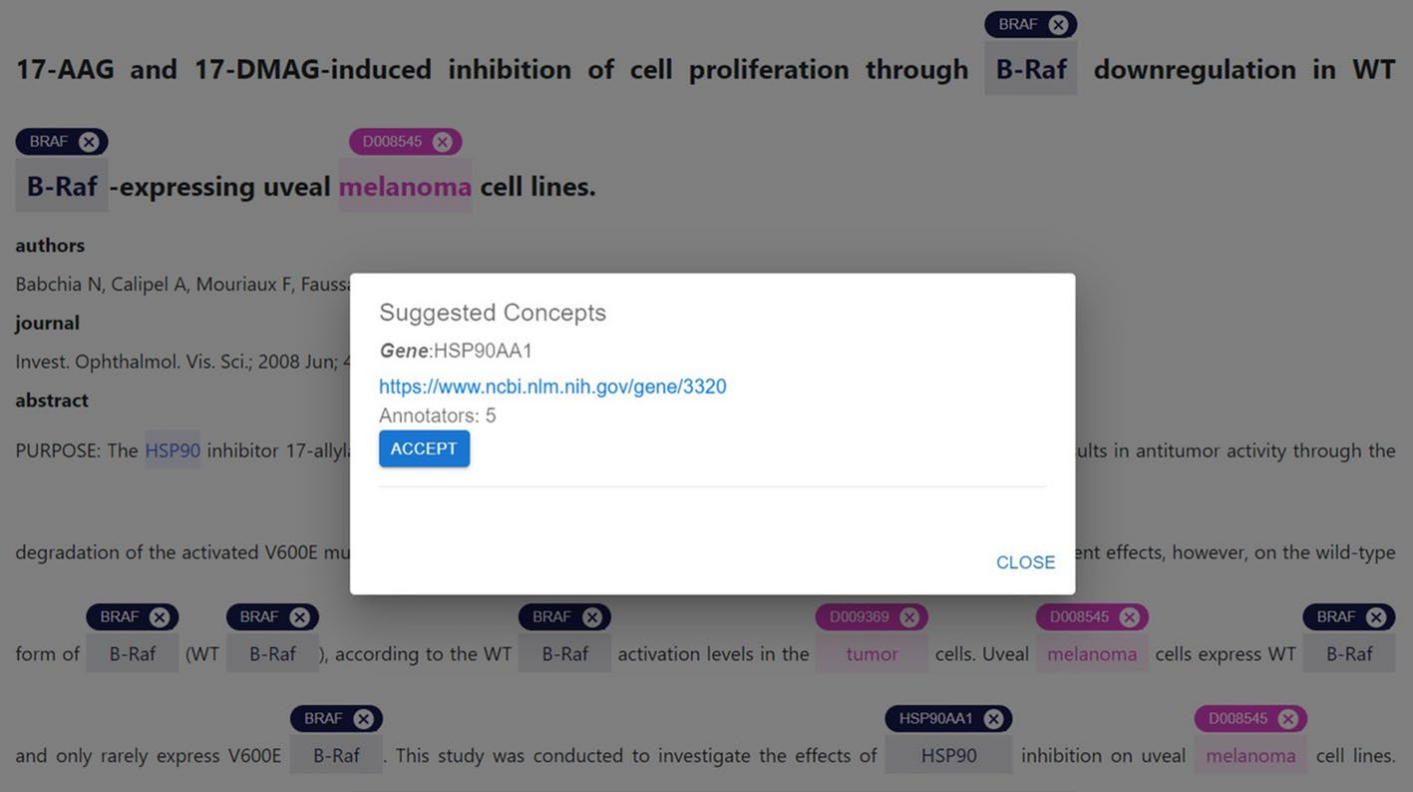
Linked concepts suggestions. The suggestion modal provides a list of concepts linked to a specific mention by the other annotators of the document. For each concept, the related information is provided; the user can link the suggested concept(s) by clicking the *Accept* button associated with the desired concepts

*The documents web page. * The user can keep track of the collection’s annotation state via the documents web page, accessible by clicking on the *Documents* button under each collection’s set of information. The documents web page, illustrated in Fig. 13, contains a dynamic table where, for each document, general information is provided—e.g., the ID, the batch number, and the number of annotations categorized by annotation type. Finally, the last column is the same for all the documents and allows one to visualize the text of the document, download its annotations, and, if the user is the creator of the collection, delete the document and the related annotations. In addition, it is possible to view all the annotations performed for each type, together with the related annotators. An example is provided in Fig. 14, where the overview of the relationships annotated for a document in the list is shown. Each relationship is subdivided into subject, predicate, and object components. If one of the components is a mention, the related text is reported along with the section in the text where it has been found—e.g., the abstract, the starting, and ending indices (this information is under the *location* field). If one of the components is an ontological concept—unlinked to any mention, instead, it is reported its type, its name (named as *concept*), and its URI (or ID). Finally, the number of annotators and their usernames are reported. This feature allows the user to have a complete overview of the relationship. Furthermore, the information about the annotators is an important indicator of the annotator’s expertise and the reliability of the annotation.

*Load the teammates’ annotations* The user can load a particular teammate’s annotation for a document directly from the vertical toolbar (C in Fig. 3). Once loaded, the user can copy one or more annotations from the teammate, resulting in both the user and the teammate having the same (or partial) set of annotations.

This feature has been implemented to allow the user to visualize and interact with other members’ annotations. The possibility to copy other members’ annotations facilitates and speeds up the annotation process as the user does not have to create new annotations from scratch.

*Receiving suggestions* By accessing the *Suggestion* option in the panel of a mention, it is possible to visualize the list of ontological concepts that the other annotators linked to that mention. Figure 15 shows an example of a suggestion modal. The concept type, name, URI (or ID), and the number of annotators are provided for each concept. The *Accept* button under placed a concept allows the user to assign that ontological concept to the mention; the *Close* button discards the suggestion. *MetaTron* allows users to link more than one suggested ontological concept to the same mention.

### Inter annotator agreement (IAA) and statistics

*Inter annotator agreement (IAA)* *MetaTron* implements two IAA methods: majority voting, Fleiss’ kappa and Cohen’s kappa. The first method selects all annotated annotations by more than half of the document’s annotators. In *MetaTron*, viewing and editing the annotations selected through majority voting is possible. The majority voting-based annotations have two goals: (i) providing the user with information about the most frequent annotations, and (ii) facilitate and speed up the annotation process. The user can copy all the annotations selected via majority voting and edit them if needed; this allows the annotator to receive an initial set of annotations, consequently saving time. It is possible to load these annotations directly from the vertical toolbar (C in Fig. 3): they can be loaded by clicking on the user called *IAA - Inter Annotator Agreement*.

Fleiss’ kappa is a statistical measure that assesses the level of agreement between two or more annotators [89]. In *MetaTron*, two Fleiss’ kappa agreement values are computed for each annotation type: one concerns the entire collection of documents, and one concerns each single document. It is possible to check Fleiss’ kappa agreement values on the *Statistics* web page, whose details are provided in the paragraph below.

Cohen’s kappa is a statistical measure ranging between $-1$ and 1 that assesses the level of agreement between two raters rating the same set of elements [90]. Similarly to Fleiss’ kappa, we provide the Cohen’s kappa for each annotation type; it is computed for the entire collection or each document, according to the users’ needs.

It is always possible to introduce new agreement functions according to the users’ needs and requirements: *MetaTron* is indeed flexible, and its modular design allows for integrating new functions and measures.

Having Fleiss’ kappa and Cohen’s kappa values provides a more complete view of annotation quality. Fleiss’ kappa can show how agreement varies with a different number of annotators, while Cohen’s kappa offers a more specific assessment between pairs of annotators. Using both coefficients ensures a more accurate evaluation of annotation reliability, considering the variety of annotators involved and the specific agreement between pairs of annotators.

**Fig. 16:**
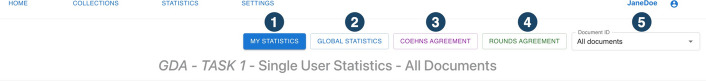
Statistics tool bar. The statistics tool bar allows to select different types of statistics. In 1 and 2 it is possible to switch between personal and global statistics. In 3 it is possible to select two annotators and get the Cohen’s kappa agreement between them; in 4 the agreement amongo multiple annotation rounds is provided; in 5 it is possible to select one document and check its statistics

**Fig. 17:**
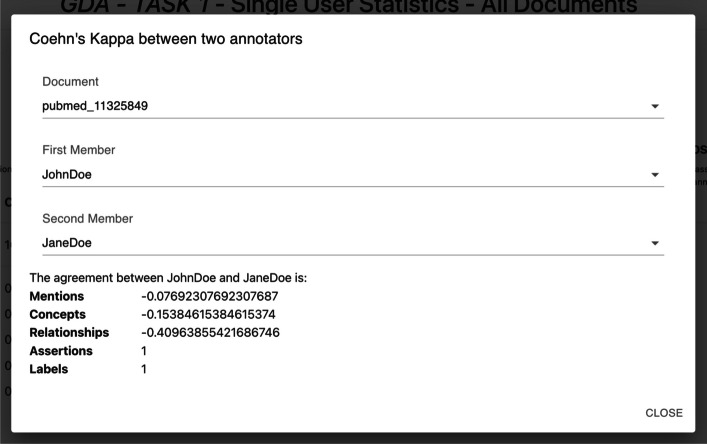
Cohen’s kappa agreement. The Cohen’s kappa agreement is provided for a pair of annotators selected by the user. The user can decide to compute the Cohen’s kappa basing on a single document or on the entire collection

**Fig. 18:**
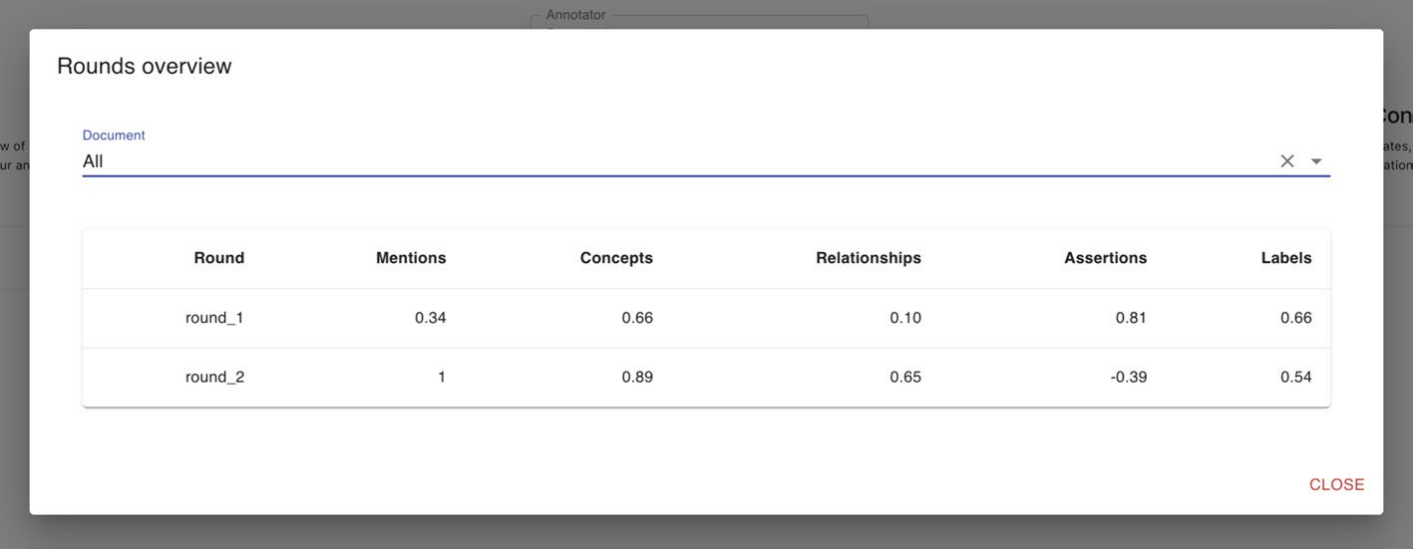
Fleiss’ kappa rounds agreement. This modal provides a table where, for each round, it is provided the Fleiss’ kappa for each type. The user can select the document or, alternatively, can check the agreement computed on the entire collection

**Fig. 19:**
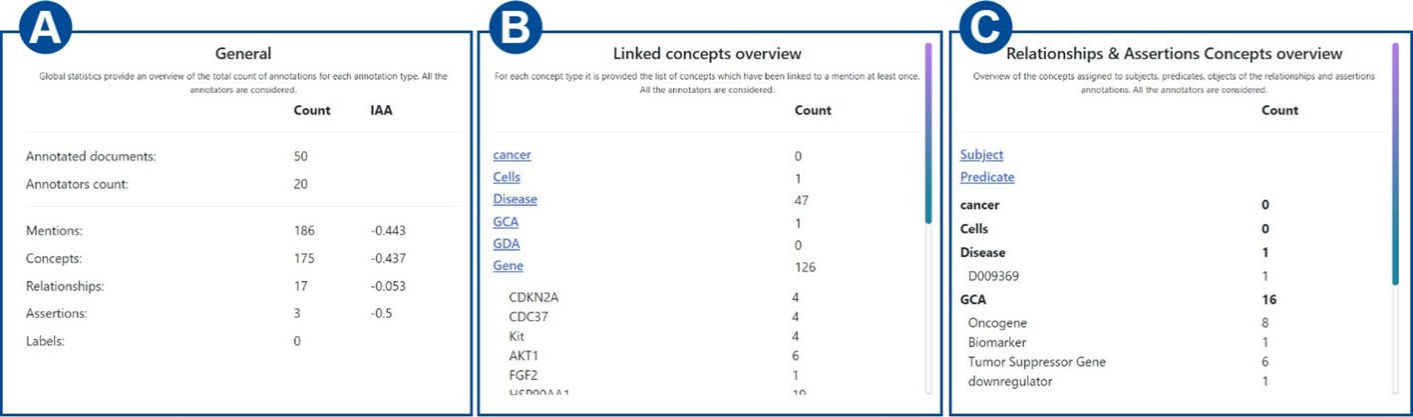
General statistics. The table at the left shows each annotation type, the total count of annotations, and the inter-annotator agreement. The second table concerns the concepts linking annotation type and for each concept type, it is possible to see how many concepts have been linked and the related name. Finally, the last table shows, for each concept type, the number of concepts (both unlinked and linked to a mention) taking part in a relationship (or an assertion) and whose role is subject, predicate, and object, respectively

**Fig. 20:**
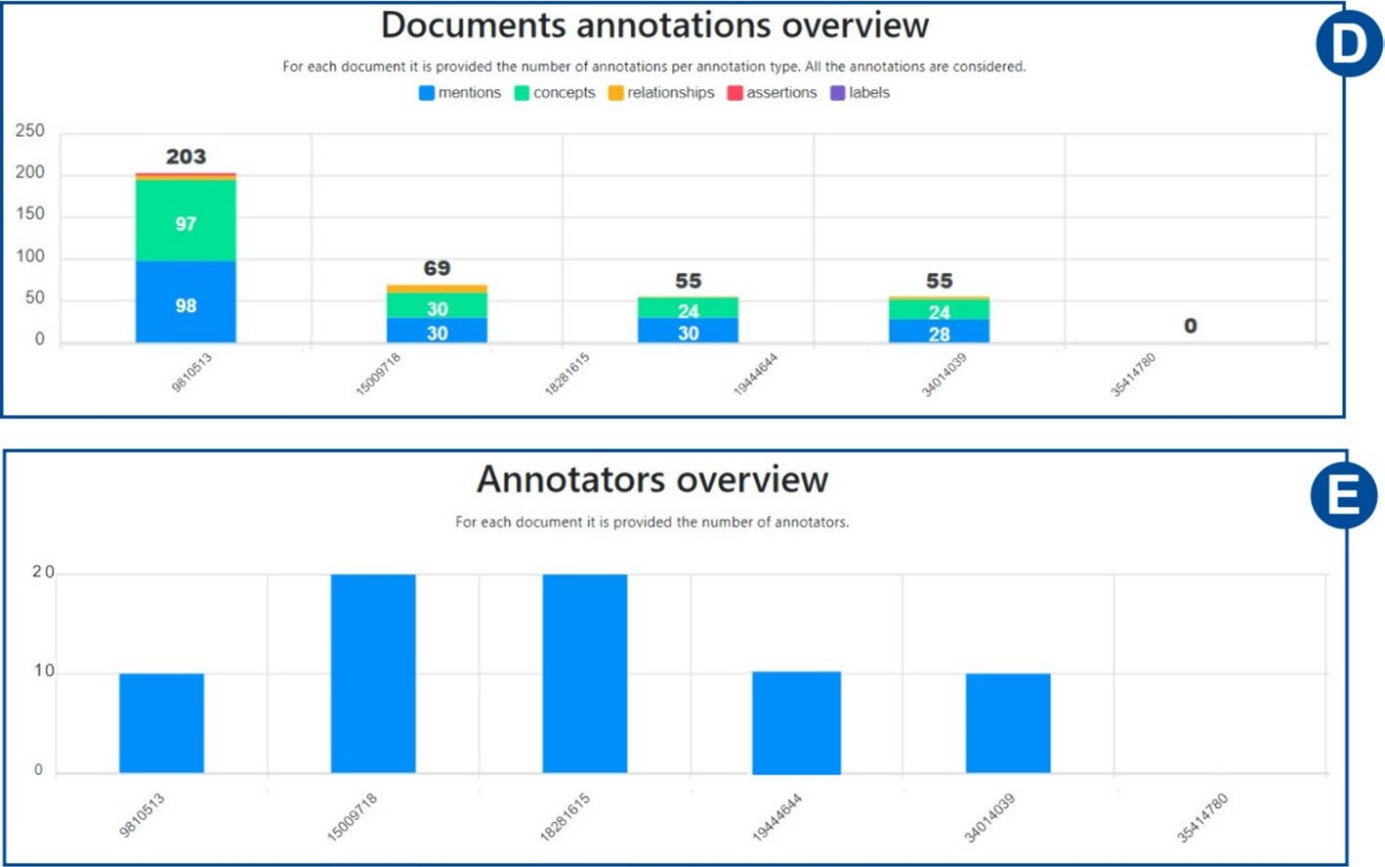
Annotations and annotators statistics. The first histogram illustrates for each document of the collection, how many annotations have been performed for each annotation type. The second instead, shows the number of annotators for each document

**Fig. 21:**
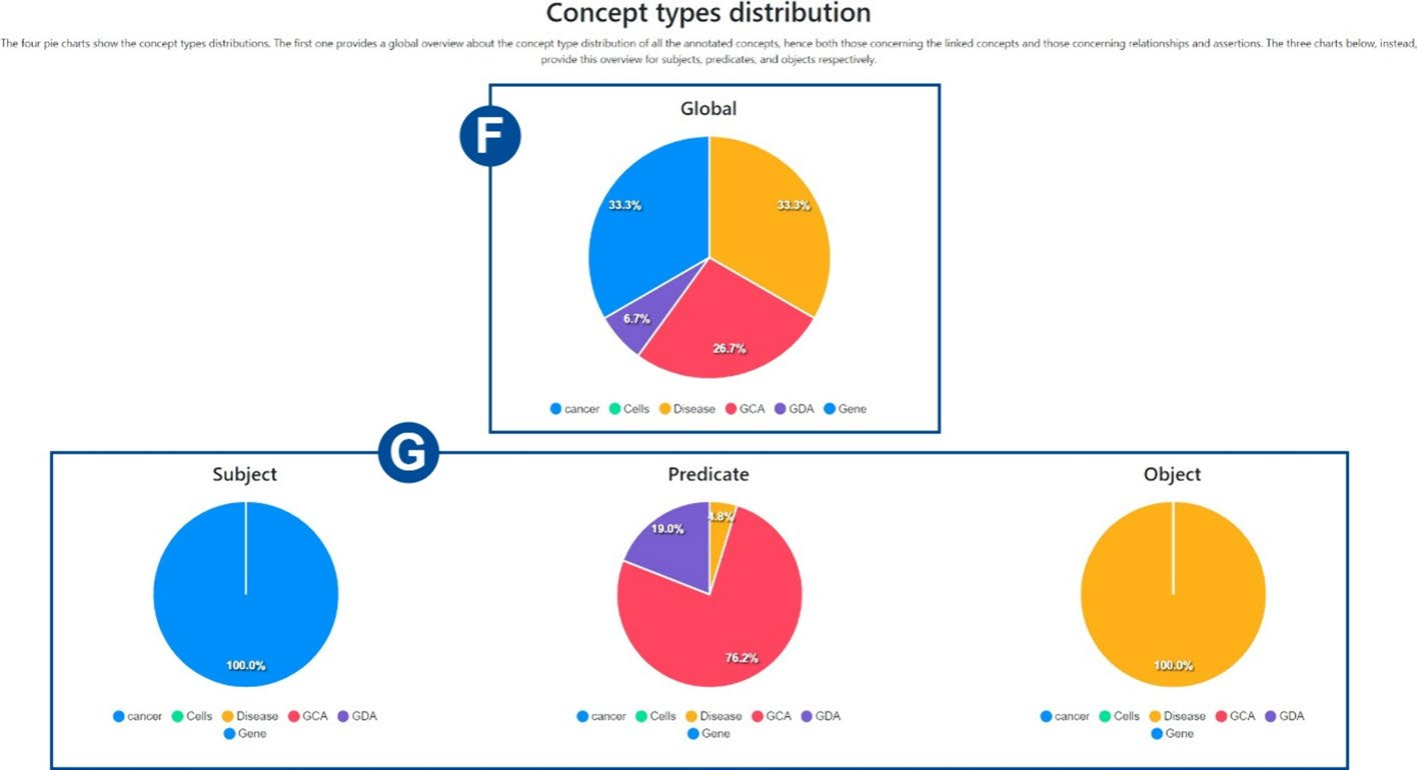
Concept types distribution. The *Global* pie chart illustrates the concepts type distribution of the concepts assigned in all the annotation types. The other pie charts illustrate the concept types distributions of the concepts (unlinked and linked to the mentions) taking part in relationships and assertions and whose role was subject, predicate, and object, respectively

*Collections’ statistics* Detailed statistics are provided on the statistics web page. *MetaTron* provides two types of statistics: (i) *personal* statistics concern the single user, and (ii) *global* statistics concern the entire set of annotators of the collection. The two buttons at the top of the page allow the user to switch between personal ((1) in Fig. 16) and global ((2) in Fig. 16) statistics. (3) in Fig. 16 allows the user to be provided with the Cohen’s kappa agreement between two users they selected and the agreement is computed for each annotation type. An example is provided in Fig. 17. (4) in Fig. 16 allows the user to be provided with the Fleiss’ kappa agreement for each annotation type on each round of annotation. An example is provided in Fig. 18: for each round it is possible to see how the agreement evolves. The text area (5) in Fig. 16) allows the user to select a document to check the statistics of—by default, the statistics concern the entire set of collection documents An example of global statistics is illustrated in Figs. 19, 20, 21. *General* statistics, (A) in Fig. 19 include the number of annotated documents, the number of annotators, the number of annotations for each annotation type, and the inter-annotator agreement computed basing on the Fleiss’ kappa measure. The *Linked concepts overview* section (B) displays the count of how many times an ontological concept has been linked to a mention. The list is subdivided into concept types. Additionally, in section (**C**), there are three lists: subject, predicate, and object lists. Similarly to the previous case, each list contains the concepts, grouped by concept type, that were annotated as a subject, predicate, or object, respectively. The *Documents annotations overview* section ((D) in Fig. 20) provides for each document of the collection the total number of annotations for each annotation type. In *Annotators per document count* ((E) in Fig. 20) the number of annotators is provided for each document. Finally, the pie chart in (F) in Fig. 21 provides a global overview of the distribution of the concept types annotated in concepts linking, relationships, and assertions annotations. The three pie charts in (G) instead exclusively concern relationships and assertions and provide an overview of the concept types assigned to subjects, predicates, and objects, respectively. *Personal* statistics show the same set of statistics, except for the *Annotators overview*—which is not considered since personal statistics exclusively concern a single user. If a document is selected instead, the *Documents annotations overview* and the *Annotators overview* are not shown since these statistics concern the entire collection of documents. It is worth noting that it is always possible to access the statistics of the documents a user annotates via the *statistics* button in the vertical toolbar, which shows both the personal and the global statistics.

## Results

This section compares a subset of the annotation tools illustrated in Fig. 1. The online tools selected for comparison are *TeamTat*, *MedTAG*, *LightTag*, and *MetaTron*. The offline tools instead are *MetaTron (dockerized)*, *INCEpTION*, and *brat*. While *TeamTat* and *MetaTron* target the biomedical domain, the other annotation tools are general purpose.

We provide a *qualitative comparison* where we outline the core functionalities of each tool. Furthermore, we provide a *quantitative analysis* by conducting experiments to assess the performance of each tool in tasks including mentions annotations and concepts linking, and relationships annotation. In the quantitative analysis, we did not consider *MedTAG*—as it does not support relationship annotation, and *brat*—as it has not been possible to automatically test it with a web agent.

We planned to include in our comparison also *tagtog* [50], however, as of May 2023, the online version of *tagtog* did not allow us to add new documents to a collection; as a consequence, it has been impossible to qualitatively and quantitatively assess its performances. Furthermore, *LightTag* does not support entity linking, hence we cannot link a concept to a mention, we can only tag the mention with a concept type. However, we included this tool in the quantitative comparison as it is one of the newest tools of the past years and allows for relationships annotation.

In the last section, we describe a user study conducted on two tasks, namely GCA and GDA. GCA focuses on the annotation of relationships where subject and object are mentions and the predicate is an ontological concept. GDA focuses on the annotation of assertions. The user study involved 10 PubMed abstracts per task annotated by three experts in the biomedical domain. All the users were initially provided with the automatic annotations performed by AutoTron. We analyzed the results *quantitatively*, measuring the agreement among the annotators, and *qualitatively* via a questionnaire involving the annotators’ experience. Finally, we studied how AutoTron impacted on the annotations analyzing how many annotations generated automatically have been updated, removed, added or confirmed.

### Qualitative analysis

Figure 1 illustrates an overview of the features characterizing a set of annotation tools. In the qualitative analysis we compare: *MetaTron*, *MedTAG*, *TeamTat*, *brat*, *LightTag*, and *INCEpTION*. The qualitative comparison is based on our direct experience with all these tools.

*MetaTron* is the unique tool that fully satisfies 23 of 24 criteria—active learning, and built-in prediction is only partially satisfied; specifically, it is the unique tool that satisfies the connection to ORCID (for login purposes) (F14) external libraries integration (F15). We see that *TeamTat*, and *INCEpTION* are the most complete tools: they fully satisfy 17 and 19 criteria, respectively, 5 criteria and 1 criterion, respectively, are only partially satisfied, and the remaining are not satisfied at all. Conversely, *LightTag*, is the least complete: 13 criteria are fully satisfied, 3 are partially satisfied, and 8 are not. Among the tools we compared *MetaTron* with, *LightTag* is the unique one without source code available (T2), does not show the date of the last version (or commit) (T1), does not allow for modification and redistribution (T6), is not free of charge (T7), and does not support ontologies (F4). *brat*, instead, is the unique tool that does not support document-level annotation; hence, it is impossible to associate one or more classes to a document. Finally, only *TeamTat* fully supports active learning (F5), and only *TeamTat* and *MedTAG* support the annotation of PubMed abstracts. Online availability (T3) is satisfied by *TeamTat* and *LightTag*; *INCEpTION* and *brat* are available offline—the online demo is available for *INCEpTION*, and *MetaTron* and *MedTAG* are available online and can be locally installed. Online tools are usually easier and faster to configure than locally installable ones: online tools usually require the user to upload a set of documents in predefined formats and a schema for the annotation, which usually includes the definition of the labels to classify the documents, or the definition of the entity types to associate. However, online tools might suffer from network delays that may occur when a large amount of data is uploaded/downloaded. Offline tools guarantee isolation and preserve data privacy; simultaneously, their installation might be difficult, and the configuration is time-demanding for someone with little technical expertise.

As of our experience with the compared tools, the offline tools—*brat* and *INCEpTION*, were the least intuitive and required the most time to be configured. In particular, *INCEpTION* required a deep study of the documentation as the notion of annotation layers that characterize the tool is not intuitive. On the other hand, *TeamTat* has the fastest and easiest configuration: it requires the definition of a collection and the upload of a set of documents. *LightTag*, together with the set of documents to annotate, required the user to define one or more annotation schemas and relation schemas: the former allows to define the tags to associate to the entities, and the classes to classify the documents with, while the latter allows user to specify the relation types—i.e., the labels to be assigned to the edge between two entities of the relationship. *MedTAG* instead requires uploading a set of documents, labels for the document-level annotation, and a set of ontological concepts.

The compared tools report substantial differences in the supported document formats: *MetaTron*, *INCEpTION*, and *TeamTat* are the unique tools supporting the upload of PDFs and TXT files. *MetaTron*, *MedTAG*, *LigthTag* support JSON and CSV. *TeamTat*, *MetaTron*, and *MedTAG* allow the user to upload PubMed abstract. Only *MetaTron* allows the user to specify a DOI and annotate the related abstract extracted from Semantic Scholar or OpenAIRE. In this respect *MetaTron* is the only tool that supports all the aforementioned formats and is integrated with three different APIs to abstracts upload.

We analyzed the annotation procedure for what concerns: labels annotation, mentions annotation, concepts linking, and relationship annotation. All the analyzed tools implement mention annotation via drag and drop, except for *MedTAG* where mentions are selected by clicking on each token composing the mention. *LightTag* is the only tool that allows users to perform entity tagging and does not support entity linking. *MetaTron* is the unique tool providing three modalities to select mentions, enhancing the annotation experience. In all the other tools, the concepts linked to a mention are always selected, specifying the related type and URI or name. Also, label annotation is similar in all the examined tools and can be achieved by clicking on the labels to be associated with the document. Relationships annotation may vary depending on the examined tool: *LightTag* and *TeamTat* for example, support n-ary relationships; in *MetaTron* instead, a relationship always has three components, and only one of them must be a mention annotated in the document. In *INCEpTION* and *brat*, instead, the source and the target in the relationships must be mentions. *MetaTron* is the most versatile tool among those described, as in a relationship, subject and object are not required to be mentions in the text. Additionally, *MetaTron* is the only tool to propose assertions annotation, not necessarily tied to sentences in the text. In this respect, the availability of document-level annotations is a relevant feature for *MetaTron* as, according to [10], the most adopted biomedical annotation tool does not implement this feature.

We compared *MetaTron* and the tools in terms of collaborative features and agreement; *MetaTron, MedTAG, TeamTat, INCEpTION and LightTag* support the collaboration between multiple annotators. Specifically, *TeamTat* supports multiple rounds of annotation and provides the annotators with agreement and disagreement between the annotators, as well as disagreement resolution. *LightTag* implements task management features, assigning tasks to different groups of annotators based on specific needs—e.g., language, and allowing project managers to keep track of annotations and agreement. *tagtog* implements user roles and allows for the definition of a set of custom annotation guidelines. In this respect, *MetaTron* implements different annotation rounds and provides some additional collaborative features to facilitate and speed up the annotators’ work. Specifically, it allows annotators to copy other members’ annotations, and the annotation with the highest agreement is computed via majority voting. In addition, *MetaTron* implements annotation suggestions: given a mention, users can see what the concepts assigned by the other annotators are and select one of them accordingly, depending, for example, on how many users have linked a specific ontological concept. For each annotation performed, the user can keep track of the number of users who performed the same annotation and change it accordingly. In *MetaTron*, the collection’s creator can keep track of the annotation progress and is responsible for selecting the annotators of each round. All the users can see the entire sets of annotations of each collection document and the related annotators. *MetaTron* is the unique tool providing different agreement measures (it implements both Fleiss’ kappa and Cohen’s kappa) computed on the entire collection or on single documents.

*brat* and *INCEpTION* are included in our comparison even if they do not target the biomedical domain.

### Quantitative analysis

To compare the performance of the selected manual annotation tools, we conducted a series of experiments on two different tasks: concepts linking, and relationships annotation. We did not treat mention annotation as a separate task because the annotation method was the same across all the annotation tools. Our experiments concerned the time elapsed and the number of clicks required to annotate a collection of the same 15 documents.

To evaluate the performances of the selected tools we relied on Selenium,^4^ an open-source testing framework used to automate web browsers. We designed four web agents, one for each annotation tool—the same web agent has been used for the two instances of *MetaTron*, and we used them to simulate the concepts liking and relationships annotations task on a collection of 15 abstracts extracted from PubMed. In order to simulate the annotator activity, we selected abstracts of various lengths; the mentions, the linked concepts, and the relationships have been extracted using *AutoTron*. Overall, *AutoTron* extracted 94 mentions and 71 relationships; specifically, for each abstract, a minimum of 2 mentions and 1 relationship, and a maximum of 13 mentions and 9 relationships were found. Each mention has been linked to exactly one concept. Variable delays were introduced based on the examined tool to prevent errors caused by server overload and to allow sufficient time for request processing. To provide a fair analysis, we treated online tools and offline tools separately, as the performance of online tools is influenced by the server hosting the application, while offline tools rely on the individual machine running the test.

**Table 1:** Overview of the time spent to perform mentions annotation (MA), concepts linking (CL), and relationship annotation (RA) on a set of 15 documents. The reported value of average (AVG), standard deviation (STD), median (MED), and 5*th* and 95*th* percentiles refer to the time spent annotating 15 documents 50 times

		MA + CL + RE				
		AVG	STD	5th	MED	95th
Online	MetaTron	**638**.**73**	1.67	636.97	**638**.**22**	642.49
	TeamTat	842.93	0.76	841.67	843.03	844.18
	LightTag	661.13	1.23	659.35	660.85	663.07
Offline	MetaTron	**642**.**39**	1.30	640.35	**642**.**28**	644.54
	INCEpTION	704.85	2.14	701.53	705.12	707.33

The boldface values represent avg and median results of the tools with the best performances, i.e., the lowest time taken to annotate 15 documents 50 times

We conducted an analysis concerning the time spent annotating the entire collection. The results are shown in Tables 1 and 2. We compared the annotation tools on the average (AVG), the median (MED), standard deviation (STD), the 5*th* and the 95*th* percentiles of the time required to annotate the entire collection 50 times. We studied the time taken to perform two tasks: (i) performing an entire annotation, which comprehends mentions annotation (MA), concepts linking (CL), and relationships annotation (RA); and (ii) annotating the mentions (MA) and linking the concepts (CL). We remark that *LightTag* does not support concept linking, it allows only to tag the mentions with concept types.

Looking at the results achieved by the online tools in the entire annotation (Table 1), we see that *MetaTron* is the most efficient tool in terms of average time, as *MetaTron* required 638.73 s on average (while *TeamTat* requires 842.93 s). *LightTag*’s performance falls between *TeamTat* and *MetaTron*. However, *TeamTat* does not reveal significant fluctuations during the 50 annotation rounds; this means that it is a tool with good stability that performs well during long annotation sessions. The substantial difference between the performance of *TeamTat* and those of *MetaTron* should be attributed to how the annotation is performed: we noticed *TeamTat* needed longer delays to save each annotation correctly. These aspects might not be visible to a human annotator who performs slower than the web agent; as such, a human annotator will take more time than an automatic one to annotate the selected set of documents. In the comparison involving the offline tools, the dockerized instance of *MetaTron* achieved better results than *INCEpTION*, as *MetaTron* required 642.39 s on average and *INCEpTION* 704.85.

**Table 2:** Overview of the time spent to perform mentions annotation (MA) and concepts linking (CL) on a set of 15 documents. The reported value of average (AVG), standard deviation (STD), median (MED), and 5*th* and 95*th* percentiles refer to the time spent annotating 15 documents 50 times

		MA + CL				
		AVG	STD	5th	MED	95th
Online	MetaTron	**329**.**25**	1.53	327.13	**328**.**96**	331.93
	TeamTat	446.33	1.34	444.28	446.53	448.53
	LightTag	412.61	1.92	409.48	412.68	415.65
Offline	MetaTron	330.35	1.37	327.98	330.28	331.96
	INCEpTION	**326**.**85**	1.91	323.60	**327**.**00**	329.92

The boldface values represent avg and median results of the tools with the best performances, i.e., the lowest time taken to annotate 15 documents 50 times

For what concerns the second task, mentions annotation and concepts linking, whose results are shown in Table 2, similarly to the previous case, the best online tool is *MetaTron*, which required 329.25 s on average while the one that requires the highest average time is *TeamTat*, that required 446.33 s. Also in this case the performances of *LightTag*, 412.61 s, fall between those of *MetaTron* and *TeamTat*. *INCEpTION* and *MetaTron (offline)* achieved comparable performances. In both the analyzed task, all the tools had a standard deviation lower than 2.5. The 5*th* and 95*th* percentiles indicate that in all the examined tools all the computed times are uniformly distributed around the median.

The online and offline instances of *MetaTron* achieved similar performances in both tasks. This can be attributed to the absence of any differences between the code running locally and the code of the online instance. Furthermore, the server hosting *MetaTron* was underutilized when we ran the automatic agents, leading to performances comparable to the docker-based instance. It is notable the case of the *INCEpTION*: it achieved the worst performances in the first task (MA + CL + RE), and the best in the second one (MA + CL). This aspect points out that the annotation of relationships is the most expensive in terms of time, while *INCEpTION* is the most efficient tool in mentions annotation and linking. *MetaTron* and *TeamTat* present similar behaviors: the average time taken in the second task is half the time taken in the first: this indicates that annotating the relationships takes the same amount of time required to annotate the mentions and link them to the concepts. Conversely, in *LightTag*, mentions annotation and linking require more than half of the time: this is partially related to the implementation of the web agent in the relationship part, and partially depends on how mentions selection and linking have been implemented in the tool.

**Table 3:** Overview of the number of clicks required to perform the annotation of 15 documents in the two selected tasks: mentions annotation and concepts linking (MA + CL) and an entire annotation—mentions annotation, concepts linking, relationship annotation (MA + CL + RA)

	MA + CL	MA + CL + RA
MetaTron	485	1028
TeamTat	500	1423
LightTag	327	611
INCePTION	391	746

We analyzed the number of clicks performed to annotate the collection. The results include the count of clicks required to annotate every single document of the collection and the clicks necessary to change the document. The results are reported in Table 3. We see that *MetaTron* and *TeamTat* are the tools that require the highest number of clicks to annotate the collection of 15 documents. This aspect is motivated by how the tools implement relationship annotation. In both the aforementioned tools, to annotate a new relationship it is required to set the two mentions and select the predicate and the associated ontological concept: all these actions makes the total number of clicks increase. *LightTag* instead, is the most efficient, however, it does not support entity linking, and this aspect motivates the lower number of clicks with respect to the other tools, since only the concept type—i.e., gene or disease, is required. The annotation of relationships in *LightTag* is the fastest compared to the other tools; the first reason is that *LightTag* requires the user to provide a schema configuration for relationship annotation and this allows the user to save clicks and time; in addition, in *LightTag* each mention/concept composing the relationship is selected by dragging and dropping it into a box hosting the relationship components, and this allows the user to save clicks—a drag and drop action is performed in a single click. *INCEpTION* is most efficient after *LightTag*, and requires a half of the clicks compared to *TeamTat*.

### Discussion

The qualitative and quantitative analyses conducted to assess the performances of *MetaTron*, *MedTAG*, *TeamTat*, *LightTag*, *brat*, and *INCEpTION* allowed us to draw some conclusion.

The results deriving from the analyses showed that *MetaTron* emerges as a competitive annotation tool in the biomedical domain, as it is the only one that fulfills all the analyzed features and achieves the best results in terms of time spent in the annotation of a set of documents. *MetaTron* provides an environment where one or more annotators can collaborate in annotating documents in five different ways, both at the document level and mention level and can leverage automatic annotations to expedite the annotation process. Additionally, *MetaTron* is open-source, free of charge, and supports a wide range of input and output formats. The tool is released as an online and offline instance, making *MetaTron* a versatile tool that can be adapted to different needs and use cases. The online instance of *MetaTron* is valuable to test its features, take advantage of *AutoTron*’s automatic annotations, and collaboratively annotate PubMed, Semantic Scholar, and OpenAIRE abstracts; the offline release guarantees data privacy and allows users to locally deploy *MetaTron* and share the tool with a controlled number of users. In addition, the offline tool is useful when dealing with large volumes of data that would require a significant amount of time for uploading.

### User study

The user study consisted of sentence-level tasks for Gene-Cancer Associations (GCA) and document-level tasks for Gene-Disease Associations (GDA). The GCA task required annotating relationships where the subject and object, representing gene and cancer mentions, respectively, are involved. The predicate corresponds to one of the following ontological concepts: (i) biomarker, indicating whether the gene associated with the disease is altered in conjunction with the disease; (ii) tumor suppressor, indicating whether the gene plays a role in preventing the disease; and (iii) oncogene, indicating whether the gene promotes the progression of the disease.

The GDA task encompassed annotating assertions where the subject, predicate, and object are ontological concepts unrelated to specific textual mentions. The subject and object represent gene and disease, respectively. At the same time, the predicate is categorized into one of the following concepts: (i) biomarker, (ii) genomic alteration, indicating a connection between a genomic alteration and the gene associated with the disease phenotype, and (iii) therapeutic, signifying the gene’s therapeutic role in ameliorating the disease.

We chose ten pertinent PubMed abstracts for each task, which three experts in the biomedical domain annotated. To streamline and expedite the annotation process for the annotators, we furnished them with automatic annotations generated by running *AutoTron* on each document.

The annotators performed two annotation rounds for each task. In round 1, the annotators had to annotate each document relying exclusively on the auxiliary information provided by the automatic annotations. The users were allowed to add new mentions, link concepts to them, and add new relationships. However, they could not rely on collaborative features to annotate the documents—i.e., the annotators could not check other members’ annotations or documents’ statistics. In round 2, instead, the annotators were asked to rely on the collaborative features of *MetaTron* to update their annotations: as a consequence, they had access to other members’ annotations and the annotation obtained via majority voting. At the end of the annotation rounds, we provided all the annotators with a questionnaire with 15 questions about their annotation experience.

In the following sections, we analyze how the annotations change after each round and how the collaborative features impact the agreement among annotators, we summarize the answers to the questionnaire, and finally, we study the quality of the annotations of *AutoTron* as a means to speed up and facilitate annotators’ work.

*Quantitative results* In Table 4, we report the Fleiss’ kappa agreement among the annotators for each task after each round. Specifically, we provide the agreement computed on concepts and relationship annotations for the GCA task. Instead, we provide the agreement computed on assertions annotation for the GDA task. Our goal is to investigate the extent to which the presence of annotations from other annotators impacts the work of the annotators. The highest agreement has been achieved by concept annotations in all the rounds. At the end of round 1, the agreement obtained in relationships (GCA) and assertions (GDA) annotation is negative, indicating that there is no agreement among the annotators. At the end of round 2, the agreement on concept annotations did not change from round 1, while the agreement on relationships increased from $-$0.2179 to $-$0.0872 and on assertions from $-$0.0364 to 0.2490. These results confirmed that the collaborative features provided by *MetaTron* play a key role in improving the results of the annotation process.

**Table 4:** Overview of Fleiss’s kappa agreement at the end of each round. The agreement has been computed for concepts, relationships, and assertion annotations for each task’s entire set of documents

	GCA		GDA
	Concepts annotation	Relationships annotation	Assertions annotation
Round 1	0.3312	− 0.2179	− 0.0364
Round 2	0.3312	− 0.0872	0.2490

*Qualitative results* At the end of round 2, each annotator compiled a questionnaire concerning their experience using *MetaTron*. The questionnaire consisted of 15 questions about annotation experience, GCA and GDA tasks, and collaborative experience, with responses ranging from 1 to 5.

About the annotation experience, the annotators identified the annotation of mentions, concepts, relationships, and assertions as a straightforward process (all the questions about the complexity of annotations received scores equal to 1 and 2). Only one annotator needed to contact the developers to clarify how to annotate. All the annotators agreed that the automatic annotations generated via *AutoTron* are a useful starting point and contributed to speeding up and facilitating the annotation process. All the annotators considered *MetaTron* had a positive annotation experience and will use *MetaTron* in the future.

Considering task complexity, all the annotators found the GCA task more complex than the GDA one. Two over three annotators assigned a score equal to 3 in the complexity of the GCA task, while only one assigned 4. In the GDA task, instead, the annotators assigned 2, 3, and 4.

One relevant aspect is the annotators’ collaboration. All the annotators found *MetaTron* effectively supports collaboration among annotators. Specifically, all the annotators agreed that the possibility of copying one or more annotations from another annotator is an important feature that speeds up the annotation process. One annotator found useful the annotation generated via majority voting (score equal to 4), while the other two annotators assigned a score equal to 3 hence this annotation did not play a key role in their annotations. Finally, two out of three annotators admitted that those of the other annotators did not significantly influence their annotations. The remaining annotator, however, made numerous changes to the performed annotations in round 2. Two out of three annotators found it necessary to discuss their annotations and determine which relationship’s predicate to apply. This highlights the difficulty of the proposed tasks.

**Fig. 22:**
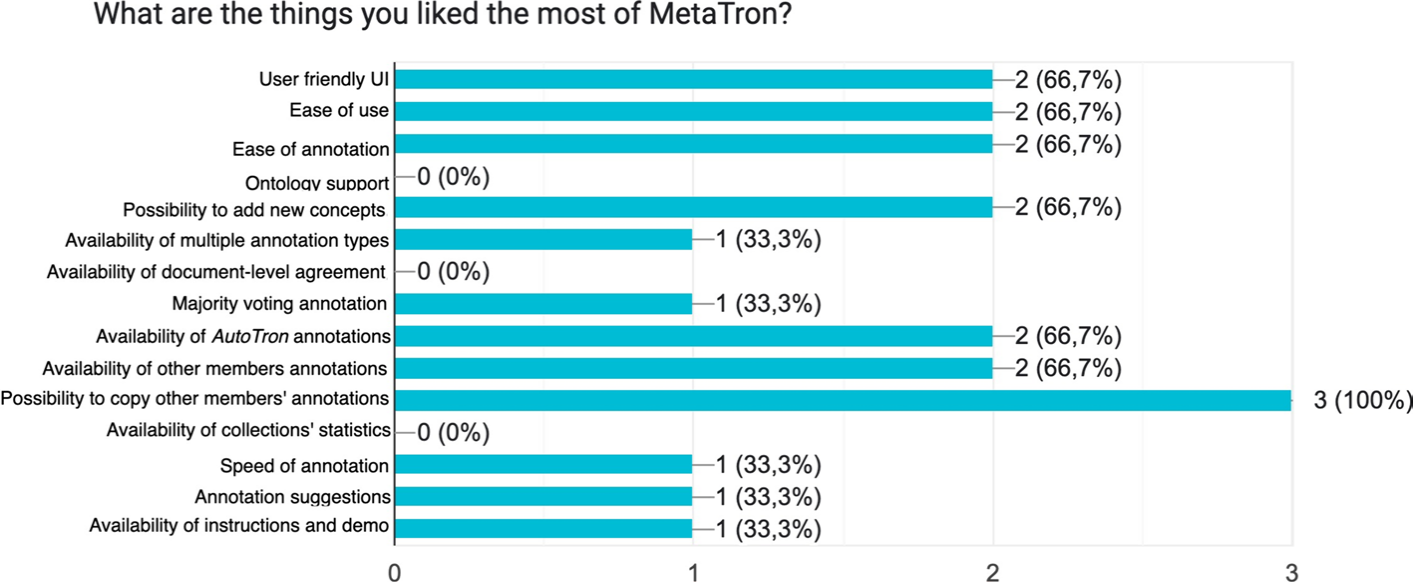
*MetaTron* features qualitative analysis. The overview concerns the features the annotators considered important to perform the two annotation tasks. The most significant features concern the collaboration among multiple annotators, while the least used concern the ontology support and the availability statistics

The annotators were required to point out the most useful features of *MetaTron* according to their experience. In Fig. 22 we provide the results of this analysis. According to our results, three features have not been selected, specifically ontology support, statistics and collection’s agreement availability. These three features had minimal impact on annotators’ work as they do not offer direct support in the annotations; instead, they prove their key role in providing insights into overall agreements. One annotator considers five features relevant features, while six features by two. The possibility to copy the annotations of another annotator has been considered the most valuable feature, as all the annotators agreed on its importance.

*AutoTron results* In Table 5, we report the total number of annotations at the end of each round. The first row, *AutoTron* refers to the total number of automatic annotations generated via *AutoTron* each annotator has been provided with at the beginning of round 1. Each annotator started with 228 concepts, and 57 relationships for the GCA task, and 17 assertions for the GDA task. At the end of round 1, the number of concepts increased to 235, the number of relationships increased to 115, and the number of assertions increased to 32. At the end of round 2, the number of concepts increased to 237, the number of relationships decreased to 111, and the number of assertions decreased to 24. The most significant change was identified in the GCA task for the doubled relationships at the end of round 1.

**Table 5:** Overview of the total number of linked concepts, relationships, assertions annotations identified at each round. The first row, *AutoTron*, represents the starting point for each annotator, indicating the set of automatic annotations provided

	GCA		GDA
	Concepts annotation	Relationships annotation	Assertions annotation
AutoTron	228	57	17
Round 1	235	115	32
Round 2	237	111	24

We considered the set of distinct annotations obtained at the end of round 2, and we counted how many concepts, relationships, and assertions have been updated, added to, and deleted from the automatically generated set of annotations provided for round 1. We considered an update when, in a relationship or assertion, the predicate assigned by the annotators is different from the one automatically assigned. We have a concept update, instead, when the linked concept changes.

In the GCA task, we detected that 9 concepts had been added, 2 were updated, 0 were removed, and 226 were confirmed. For what concerns relationships instead, 29 relationships have been added, 27 updated, 0 deleted, and 30 confirmed. In the GDA task, we detected that four assertions have been added, three updated, 0 deleted, and 14 confirmed.

The absence of deleted annotations confirms that *AutoTron* overall generates accurate annotations. Only in the GCA task in the relationships annotation the number of relationships added, updated, and confirmed remains the same.

Our results indicate that relying on *AutoTron* to generate a set of annotations used as a starting point is useful to facilitate and speed up the entire annotation process of the annotators. However, especially for relationships, the intervention of a human annotator is crucial for identifying and updating all existing relationships in a document. In this respect, according to the results obtained in the qualitative analyses, we see that the annotators found the GDA task easier than the GCA one: this results not only in a higher agreement but also in a lower number of updates and additions with respect to the automatic annotations.

## Conclusions

This paper presents *MetaTron*, a collaborative web-based annotation tool designed specifically for the biomedical domain. The tool facilitates the annotation of mentions, relationships, and document-level labels. It supports various document formats, including PDF, TXT, JSON, and CSV. Additionally, users can utilize the PubMed, Semantic Scholar, and OpenAIRE REST APIs to upload PMIDs or DOIs and annotate corresponding abstracts. Furthermore, *MetaTron* allows users to leverage their teammates’ annotations and incorporate annotations generated by *AutoTron* for fast annotation creation.

To ensure data privacy and limit tool usage to specific research groups, the *MetaTron* docker image enables local deployment on personal servers. Conversely, the online instance of *MetaTron* is designed for online annotation of PubMed, Semantic Scholar, and OpenAIRE abstracts, with the added advantage of utilizing AutoTron’s automatic prediction capabilities.

Noteworthy features of *MetaTron* include support for multiple ontologies, multilingual capabilities, login via ORCID ID, and the option to download annotations in JSON, CSV, and BioC/XML formats.

In our evaluation, we compared *MetaTron* to five other annotation tools, both general purpose and specifically tailored to the biomedical domain. We assessed them against 24 criteria classified into three categories: *Data*, *Technical*, and *Functionalities*. The qualitative analysis revealed that *MetaTron* fulfills almost all of the selected criteria. From a quantitative perspective, the online instance of *MetaTron* outperformed *TeamTat* and *LightTag* in terms of time elapsed and the number of clicks required. Additionally, the dockerized version of *MetaTron* achieved better results than *INCEpTION* in the task of mentions annotation, concept linking, and relationship annotation (MA + CL + RA). We conducted a user study which involved three human annotators and two tasks: relationships annotation and assertions annotation. The user study pointed out that *MetaTron* is an intuitive and easy to use tool. The collaborative features have been of great assistance, enabling annotators to enhance the accuracy of their annotations and improve agreement. The possibility of using *AutoTron* to automatically annotate documents, and to copy other members’ annotations has proven to be one of *MetaTron*’s most valued features, streamlining and facilitating the annotation process. In summary, *MetaTron* presents itself as a compelling annotation tool for the biomedical and bioinformatics community, providing collaborative and interactive features that can effectively streamline the annotation process. With a commitment to ongoing maintenance and a notable emphasis on relation annotation, often overlooked by other annotation tools, we think that *MetaTron* represents one of the highly recommended options for researchers in these domains.

As future work, we plan to integrate more use cases for built-in automatic predictions to allow *MetaTron* to widen to other domains of applications for automatic annotation of text. Moreover, two functionalities deserve to be implemented: the first one is to introduce a new annotation type, which is entity tagging, and let the user associate to a mention a concept type instead of the concept itself; then, we plan to implement discontinuous mentions allowing the user to be more accurate letting them decide which tokens compose the mention. About the support for the ontologies, we plan to introduce the possibility of (i) uploading the full ontologies directly from the related files and (ii) automatically suggesting the concept to associate to a mention relying on the textual and semantic similarity between the mention and the concepts. These features would support the user in speeding up the upload and the selection of the concepts. Finally, we plan to introduce in *MetaTron* some large corpora of documents that one or more members can annotate: this would provide the community with a tool already configured and ready-to-use and would promote the analyses of annotators’ behavior on well-known datasets.

## Availability and requirements

- Project name: MetaTron
- Project home page: https://github.com/GDAMining/metatron/
- Online instance: https://metatron.dei.unipd.it
- Archived version: not applicable
- Operating system(s): Platform independent
- Programming language: Python, JavaScript, HTML, CSS
- Other requirements: Docker and docker-compose (for the dockerized version) for the offline version
- License: MIT License
- Any restrictions to use by non-academics: No

## Data Availability

The code used in this study is publicly available on GitHub https://github.com/GDAMining/metatron/. All the underlying libraries used in this work are open-source. The complete list of libraries and their versions are reported in the GitHub repository.

## Footnotes

1 https://www.djangoproject.com.

2 By accessing with the username *demo* and password *demo*, it is possible to annotate a test collection.

3 A relationship that solely consists of ontological concepts is treated as a document-level assertion.

4 https://www.selenium.dev.
